# Integrated Transcriptomic and Proteomic Analyses Reveal the Effects and Mechanisms of Glycyrrhiza Polysaccharides in Alleviating Immune Stress in Broilers

**DOI:** 10.3390/ani16172633

**Published:** 2026-08-22

**Authors:** Shuyan Wu, Boyi Dong, Dongying Weng, Jiaqi Chen, Hongzhu Jin, Xinrui Lin, Weize Qin, Shuyi Li, Haidong Du, Tiyu Li

**Affiliations:** College of Animal Science and Technology, Inner Mongolia Minzu University, Tongliao 028000, China; 15248552127@163.com (S.W.); d3129452005@163.com (B.D.); 18447024294@163.com (D.W.); cjq15149826049@163.com (J.C.); 18524937655@163.com (H.J.); 15326836969@163.com (X.L.); qwz@imun.edu.cn (W.Q.); lsy2835245597@163.com (S.L.); duhaidong1110@163.com (H.D.)

**Keywords:** Glycyrrhiza polysaccharides, broiler, immune stress, signaling pathways

## Abstract

Immune stress under intensive high density production conditions impairs broiler health and performance, highlighting the need for effective nutritional strategies. This study established an immune stress model by intraperitoneal lipopolysaccharide (LPS) injection to investigate the mechanism by which Glycyrrhiza polysaccharides (GPs) alleviate LPS-induced immune stress. A total of 240 AA broilers were randomly assigned to four groups: control (CON), GP, LPS, and GP + LPS, with six replicates per group and ten birds per replicate. During the stress phase (28 d), LPS challenge increased serum ACTH and CORT concentrations and altered several immune-related parameters; whereas, GP supplementation improved ileal morphology and reduced serum IL-1β, NF-κB, and TNF-α concentrations. Significant GP × LPS interactions were observed for IL-4, IgA, IgG, and iNOS. During the recovery phase (35 d), GP supplementation increased serum IgA and decreased iNOS, while significant GP × LPS interactions were detected for IL-1β, NF-κB, and sCD4. Notably, GP attenuated the LPS-associated increase in NF-κB. Integrated multi-omics analysis indicated coordinated regulation at transcriptional and proteomic levels. GP upregulated *FOS*, *JUN*, and *CATH3* and enriched NF-κB and Wnt signaling at the transcript level, while increasing IRF1 and CATH3 and decreasing CTNNBIP1 at the protein level, primarily involving Wnt and MAPK pathways. Joint analysis identified CATH3 as a central regulatory target. Collectively, GP alleviates immune stress by restraining excessive inflammation and modulating Wnt/MAPK signaling, thereby promoting intestinal structural recovery.

## Simple Summary

Modern high density poultry production often exposes broilers to stressful conditions that can harm their health and reduce growth, limiting production efficiency. Therefore, developing safe and effective nutritional strategies is important. This study evaluated the use of Glycyrrhiza polysaccharides as a feed additive to improve the health of broiler chickens. The results show that this additive can alleviate the inflammatory response caused by immune stress. Further omics analysis revealed that the immunomodulatory effect of Glycyrrhiza polysaccharides is achieved through the key functional molecule CATH3. Overall, this study provides a safe, green, and sustainable nutritional approach to improve broilers’ health and production efficiency.

## 1. Introduction

Broilers are a primary food source in the global poultry sector. Their favorable nutritional profile, low production cost, and rapid growth rate have made them an essential component of modern food production systems [1,2]. However, in high stocking density and intensive production systems, broilers are routinely subjected to a range of stressors during the rearing period, including environmental, pathological, and psychological challenges [3,4].

Under concurrent exposure to multiple stressors, the immune system remains in a highly activated state and engages the hypothalamus-regulated pituitary–adrenal axis and sympathetic–adrenal medullary axis to release catecholamines and glucocorticoids. These responses sustain immune cell activation and drive excessive cytokine production, ultimately resulting in immune dysfunction [5,6]. Consequently, immune stress is considered a key factor underlying abnormal behavior, metabolic dysregulation, and reduced production performance in animals [7].

To mitigate the adverse effects of immune stress, antibiotics are commonly used in animal production; however, long-term use can facilitate the spread of antibiotic-resistant bacteria and lead to drug-residue concerns [8]. Given the increasing prevalence of countries that have banned antibiotics as feed additives, the identification of safe and efficacious alternatives has gradually become a core research priority [9]. Animal nutritionists commonly employ dietary interventions, such as the addition of exogenous enzymes, botanical extracts, or probiotics to livestock and poultry diets, to mitigate the detrimental effects of immune stress [10]. Among these options, plant extracts have been widely explored as potential replacements for antibiotics due to their natural composition, absence of residues, and biological activities, including immunomodulation, maintenance of intestinal homeostasis, antibacterial actions, and inflammation-modulating effects [11,12,13].

*Glycyrrhiza uralensis*, as a traditional Chinese herbal medicine, is rich in bioactive components such as polysaccharides, flavonoids and triterpenoid saponins. Due to its significant medicinal value, it has attracted extensive attention from researchers [14]. Among them, Glycyrrhiza polysaccharides (GPs) is recognized as a key bioactive constituent derived from *Glycyrrhiza uralensis* and is reported to exert diverse pharmacological functions [15]. However, current evidence is largely derived from mouse models, and studies in broilers remain limited. In our previous study, supplementing broiler diets with 1500 mg/kg GP upregulated growth-related gene expression and improved meat quality, indicating a potential role for GP in supporting growth and development in broilers [16]. Zhang et al. [17] have demonstrated that supplementing with 1% GP not only increases the proportion of beneficial bacteria (*Lactobacillus* and *Bifidobacteria*) in the cecum of broilers, but also enhances the immune function of broilers by increasing the phagocytic index of macrophages. Moreover, Zhang et al. [18], using a lipopolysaccharide (LPS)-induced immune challenge model in broilers, demonstrated that GP attenuated the LPS-driven increases in hepatic inflammatory mediators, including interleukin-1 beta (IL-1β) and interferon gamma (IFN-γ), indicating a protective effect against immune stress.

Although previous studies have indicated that GP has the potential to regulate immune function in broilers, its protective effects against LPS induced immune stress and the underlying molecular mechanisms have not yet been elucidated. Therefore, in this study, growth performance, immune function, and intestinal barrier-related parameters were measured to evaluate the alleviating effects of GP on immune stress in broilers. Meanwhile, integrated transcriptomic and proteomic analyses were conducted to further reveal its potential regulatory mechanisms. This study provides a theoretical basis for elucidating the immunomodulatory effects of GP and offers scientific support for its development and application as a plant-derived functional feed additive in broiler production.

## 2. Materials and Methods

### 2.1. Animal Management

A total of 240 healthy 1-day-old Arbor Acres (AA) broilers with similar body weights (51.21 ± 1.96 g) and an equal sex distribution were used in this experiment. Birds were randomly assigned to a 2 × 2 factorial design, with the main factors being whether GP and LPS treatment are added, resulting in a total of four groups: CON, LPS (CON + LPS), GP, and GP + LPS. The sample size for each group (n = 6 repetitions × 10) was the same. The injection time for the LPS group was 12, 16, 20, 24, and 28 days. The GP group received a dietary supplementation of 1500 mg/kg GP throughout the entire experimental period (1–35 d), as referenced from Li et al. [16].

**Figure 1:**
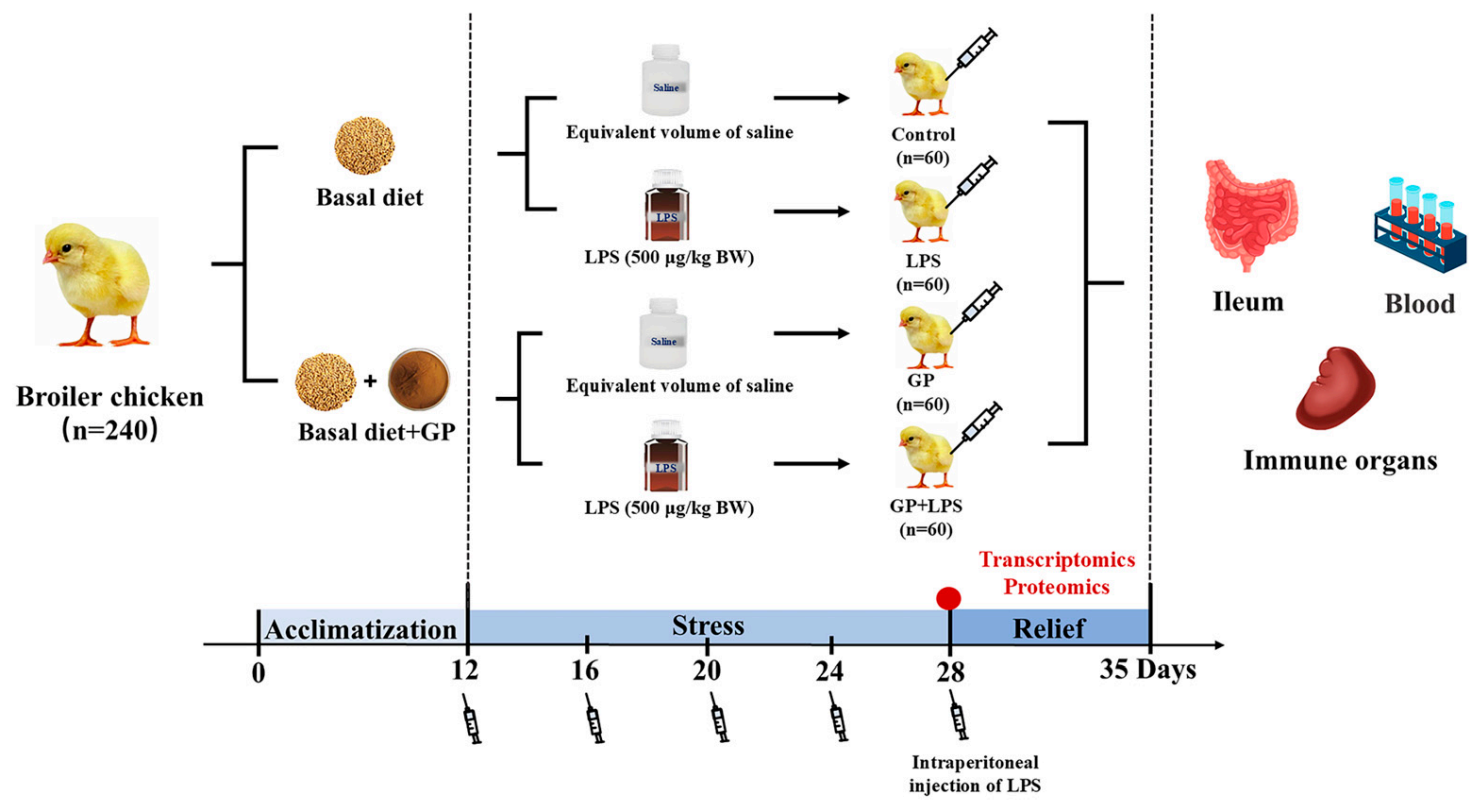
Processing of experimental broilers and flowchart of sample collection.

The LPS for the challenge in both the LPS groups was sourced from Escherichia coli (Sigma-Aldrich, St. Louis, MO, USA). The LPS dosage and route of administration were according to the method described by Jin et al. [19]. LPS (500 µg/kg BW) was dissolved in saline and injected intraperitoneally. The incidence of diarrhea and mortality were observed and recorded. The incidence of diarrhea in the control group and the LPS group was observed over a period of 12 to 28 days. The following were the criteria for judgment: 0, represents normal (solid); 1, semi-solid; 2, loose feces; and 3, watery feces [20]. Diarrhea was determined when the score was 2 or 3. Diarrhea incidence was calculated as the percentage of birds classified as having diarrhea relative to the total number of birds in each treatment. At the same time, the non-stress group received an equivalent volume of physiological saline. This experiment was divided into an initial stage (1–11 days), stress phase (12–28 days), and recovery phase (29–35 days). The specific experimental grouping method is shown in Figure 1.

The experiment was conducted in single-tier cages. In the first week, broiler chickens were maintained under continuous lighting for 24 h. Beginning in the second week, they received a 23 h light and 1 h dark photoperiod. Throughout the rearing period, ambient temperature was controlled within the range of 32–35 °C. Beginning in the first week, the temperature was reduced by 1 °C at 2-day intervals, gradually decreasing to 24–26 °C by the third week and maintained at this level until the experiment’s conclusion. Relative humidity was maintained between 60% and 70%. Throughout the experimental period, feed and water were provided ad libitum, and the birds were vaccinated according to the standard immunization schedule (Table 1). The basal diet was formulated in accordance with NRC (1994) nutrient recommendations for broilers [21] (Table 2).

### 2.2. Characterization of Glycyrrhiza Polysaccharides

**Figure 2:**
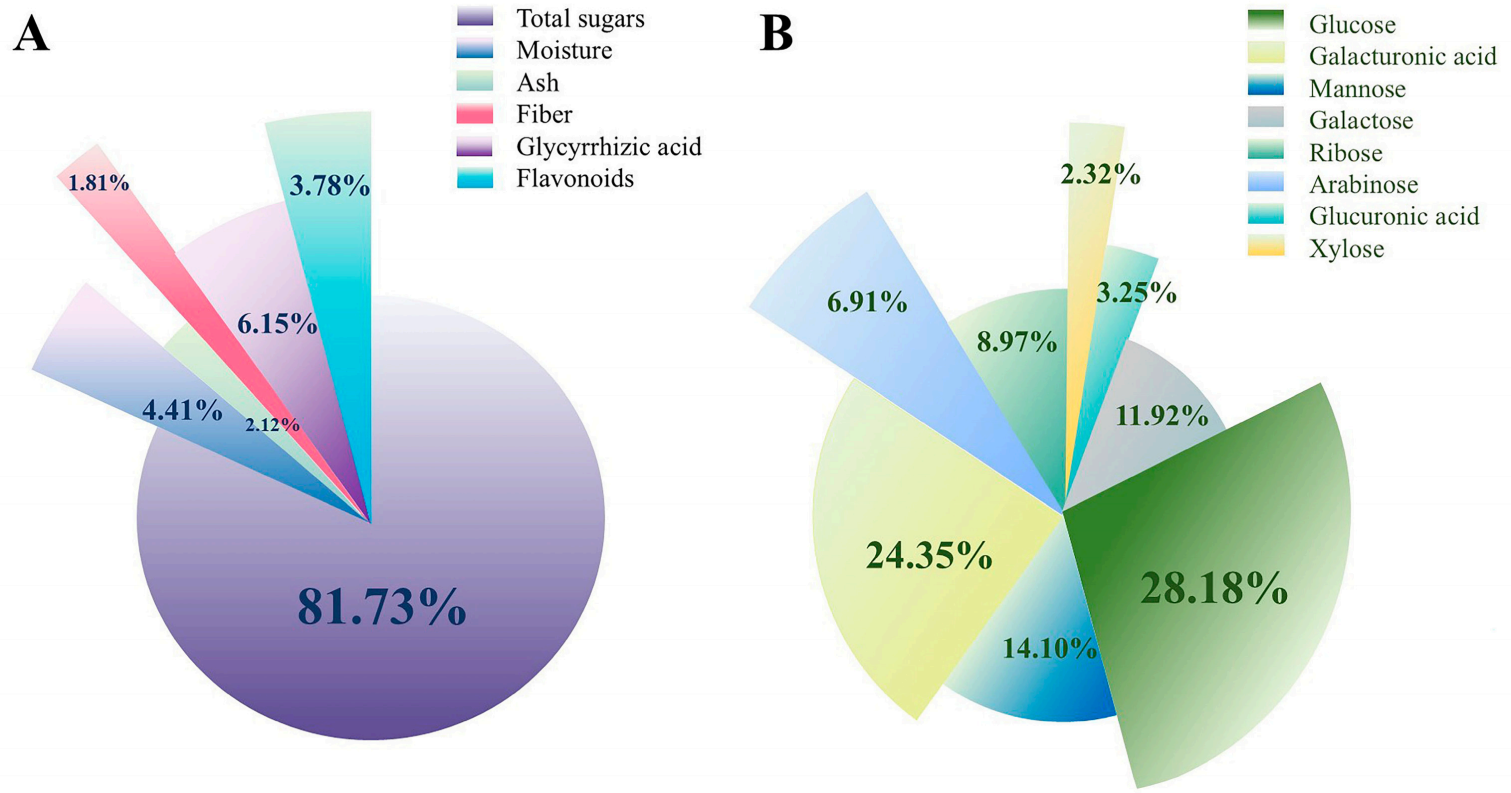
Composition analysis and monosaccharide composition of Glycyrrhiza polysaccharides (GP) extract. (**A**) Percentage of major components in GP extract. (**B**) Monosaccharide composition of GP extract in percentage.

The GP was obtained from Dongjiang Tiancheng Industrial Co. (Weinan, Shaanxi, China) with a purity of ≥80%, and the analysis results for total sugar content and monosaccharide composition are presented in Figure 2.

The total carbohydrate content of GP was determined using the phenol–sulfuric acid colorimetric method with D-glucose as the standard, according to the method of Dubois et al. [22].

The monosaccharide composition of GP was determined by high-performance anion-exchange chromatography coupled with pulsed amperometric detection (HPAEC-PAD), based on previously described methods [23]. Monosaccharides were identified by comparing their retention times with those of authentic standards and quantified using external calibration curves based on peak areas. The relative monosaccharide composition was expressed as the molar percentage of each monosaccharide.

### 2.3. Growth Performance Measurements

After feed was withheld for 12 h, in each replicate, broilers were weighed on days 1, 12, 28, and 35, and daily feed intake was recorded. Average daily gain, average daily feed intake, and feed conversion ratio were calculated for each period.

### 2.4. Sample Collection

Four hours after LPS injection on days 12, 16, 20, 24 and 28, and 12 h after the end of feeding on days 28 and 35, one broiler from each replicate group was selected to draw blood, which was close to the average weight of that group. Approximately 5 mL of blood was obtained from the wing vein, followed by centrifugation at 1400× *g* for 15 min; the resulting serum was collected and stored at −20 °C. Birds were humanely euthanized by cervical dislocation. After removing the fat, the spleen, thymus, liver, and bursa of Fabricius were weighed (accurate to 0.01 g). Additionally, a minimum of 1 g of spleen tissue was placed into a 15 mL centrifuge tube, immediately preserved by liquid nitrogen freezing, and retained for later omics-based analyses. A 5 cm segment from the mid-ileum was collected, gently rinsed with PBS to remove digesta, and fixed with 4% formaldehyde to ensure organizational integrity [24]. Histological examination confirmed that the tissue structure was well-preserved and there were no signs of autolysis.

### 2.5. Ileal Tissue Morphology

The ileum samples were transferred into 50% ethanol for dehydration for 45 min per step, and the procedure was repeated twice. The tissues were dehydrated using a stepwise ethanol gradient consisting of 70%, 80%, 90%, 95%, and absolute ethanol, after which they were embedded in paraffin wax. The tissue sections were cut at a thickness of 5 micrometers and stained with hematoxylin-eosin (H&E) and Periodic Acid-Schiff (PAS) stain. Morphological observations were performed under a microscope (Ci S, Nikon Corporation, Tokyo, Japan). Villus height (VH), crypt depth (CD), and goblet cell counts (GCs) were measured using ImageJ (v1.49).

### 2.6. Determination of Immune Organ Index

Determine the immune organ indices of the liver, spleen, bursa of Fabricius, and thymus. Calculate according to the following formula: Immune organ index (%) = 100 × Immune organ weight (mg)/Body weight (g).

### 2.7. Enzyme-Linked Immunosorbent Assay (ELISA) Analysis

On days 28 and 35, serum levels of IL-1β, interleukin-4 (IL-4), interleukin-6 (IL-6), interleukin-10 (IL-10), nuclear factor kappa-B (NF-κB), tumor necrosis factor-α (TNF-α), immunoglobulin A (IgA), immunoglobulin G (IgG), immunoglobulin M (IgM), soluble cluster of differentiation 4 (sCD4), soluble cluster of differentiation 8 (sCD8), toll-like receptor 4 (TLR4), inducible nitric oxide synthase (iNOS), corticosterone (CORT), and adrenocorticotropic hormone (ACTH) were quantified using commercially available ELISA kits according to the manufacturer’s instructions (Enzyme-linked Biotechnology Co., Ltd., Shanghai, China).

### 2.8. Transcriptomics Sequencing and Analysis

Total RNA was extracted from spleen tissues of the LPS and GP + LPS groups (three biological replicates per group) using QIAzol Lysis Reagent (Qiagen, Hilden, Germany). For transcriptome library preparation, 1 μg of RNA from each sample was used as input material.

The concentration and purity of RNA were measured using a NanoDrop 2000 spectrophotometer (Thermo Fisher Scientific, Waltham, MA, USA), with an OD260/280 ratio of 1.8–2.2, and RNA integrity was evaluated with an Agilent 5300 Bioanalyzer (Agilent Technologies, Santa Clara, CA, USA).

Library construction was conducted following the Illumina Stranded mRNA Prep Ligation protocol (San Diego, CA, USA). PolyA-containing mRNA was purified using oligo(dT) selection of magnetic particles and subsequently split into several parts averaging approximately 300 bp with a dedicated segment buffer zone. Subsequently, complementary DNA strands were generated using random hexamer primers, after which the resulting products underwent terminal repair and ligation with sequencing adapters in accordance with the established library preparation procedure. Target cDNA fragments were selected by magnetic beads and amplified through 13 cycles of PCR. The constructed libraries were quantified using a Qubit 4 fluorometer (Thermo Fisher Scientific, Waltham, MA, USA), and carry out sequencing operations on an Illumina NovaSeq X Plus system (PE150). Genes exhibiting a false discovery rate (FDR) < 0.05 and an absolute log2 fold change (|log2FC|) ≥ 1 were identified as differentially expressed genes (DEGs). Gene Ontology (GO) and Kyoto Encyclopedia of Genes and Genomes (KEGG) enrichment analyses were performed using GOATOOLS (v0.6.5) and the Python SciPy package (v1.0.0), respectively, with corrected *p*-values < 0.05 considered significant.

### 2.9. Analysis of Protein–Protein Interaction Networks

Protein–protein interaction (PPI) networks were constructed using STRING (v12.0) based on DEGs associated with the attenuation of LPS-induced immune stress by GP in broilers. Network visualization was performed using Cytoscape (v3.10.3).

### 2.10. Validation of RNA Sequencing (RNA-Seq) Results Using qRT–PCR

RNA was extracted from spleen samples by TRIzol-based isolation following the procedure reported by Livak and Schmittgen [25]. RNA concentration and purity were determined based on optical density measurements at wavelengths of 260 and 280 nm using a spectrophotometer (Thermo Fisher Scientific, Waltham, MA, USA). cDNA was synthesized using the PrimeScript RT reagent kit with gDNA Eraser (Takara Bio Inc., Otsu, Japan) in accordance with the manufacturer’s instructions. Quantitative PCR (qPCR) was carried out on an Illumina PCR system using TB Green (TB Premix Ex Taq II, Perfect Real Time, Takara Bio Inc., Otsu, Japan) according to the manufacturer’s guidelines. The thermal cycling process involves: Firstly, a 10 min denaturation treatment is carried out at 95 °C. Then, 40 cycles are conducted, with each cycle consisting of 15 s at 95 °C and 60 s at 60 °C. A melting curve analysis was then conducted. Using β-actin as the internal reference, the relative expression levels of iNOS, NF-κB p65, TLR4, myeloid differentiation primary response 88 (MyD88), lymphocyte antigen 96 (LY96), and p38 mitogen-activated protein kinase (p38 MAPK) genes in the spleen were calculated by the 2^−ΔΔCT^ method. The primer sequences are shown in Table 3.

### 2.11. Proteomics Sequencing and Analysis

For 4D label-free proteomic analysis, spleen samples from the LPS and GP + LPS groups (three biological replicates per group) were collected, and a lysis buffer containing 1% sodium dodecyl sulfate (SDS), 8 M urea, and protease inhibitors was prepared. Lysates were homogenized and sonicated in three 40 s cycles. Then, the samples were placed on ice for 30 min for cultivation. Samples were then centrifuged at 16,000 g for 30 min at 4 °C, and the supernatants were used for protein quantification with a bicinchoninic acid (BCA) Assay Kit (Pierce, Thermo Fisher Scientific, Waltham, MA, USA) following the manufacturer’s protocol.

For each sample, 100 μg of protein was brought to 100 mM triethylammonium bicarbonate (TEAB) and reduced with 10 mM tris (2-carboxyethyl) phosphine (TCEP) and incubated at 37 °C for 1 h. Then, 40 mM iodoacetamide (IAA) was added, and an alkylation reaction was carried out at room temperature in the absence of light for 40 min. Cold acetone was introduced into the samples at a volume ratio of 6:1 (acetone: sample), and proteins were allowed to precipitate at –20 °C for 4 h. The resulting pellet was recovered and reconstituted in 100 μL of 100 mM TEAB. Protein digestion was performed using sequencing-grade trypsin at an enzyme-to-protein ratio of 1:50 (*w*/*w*), with incubation at 37 °C overnight. Peptide samples were dissolved in 0.1% trifluoroacetic acid (TFA) and desalted on HLB solid-phase extraction columns. The eluates were vacuum dried and quantified with a commercial Peptide Quantification Assay Kit (Thermo Fisher Scientific, Waltham, MA, USA) following the manufacturer’s protocol.

LC-MS/MS analysis was conducted after peptide samples were loaded in LC buffer on an EASY-nLC 1200 system (Thermo, Waltham, MA, USA) coupled to a timsTOF Pro2 mass spectrometer (Bruker Daltonics, Bremen, Germany). Raw MS/MS data were processed in MaxQuant (v2.0.3.1) software for protein identification against the corresponding database (*Gallus_gallus*). Protein expression differences between groups were evaluated using the *t*-test function in R. Proteins with a fold change (upregulated) ≥ 2 or ≤0.5 (downregulated) and *p* < 0.05 were defined as differentially expressed proteins (DEPs). Quantitative and qualitative data were then subjected to downstream statistical and bioinformatics analyses. GO and KEGG pathways were screened for significant enrichment at parameters as those used for the transcriptomic analysis.

### 2.12. Statistical Analysis

Data were summarized using Microsoft Excel 2019 and statistically analyzed using SAS version 9.4 (SAS Institute Inc., Cary, NC, USA). Data from the 2 × 2 factorial experiment were analyzed using the GLM procedure, with dietary GP supplementation, LPS challenge, and their interaction (GP × LPS) included as fixed effects. When a significant GP × LPS interaction was detected, treatment means were further compared using Tukey adjusted multiple comparisons. To validate the LPS-induced immune stress model, the differences in serum ACTH and CORT concentrations between the CON and LPS groups at each sampling time point were analyzed using independent samples *t* tests. Diarrhea incidence and mortality were summarized descriptively. Differences were considered statistically significant at *p* < 0.05.

## 3. Results

### 3.1. Validation of the Repeated LPS-Induced Immune Challenge Model

**Figure 3:**
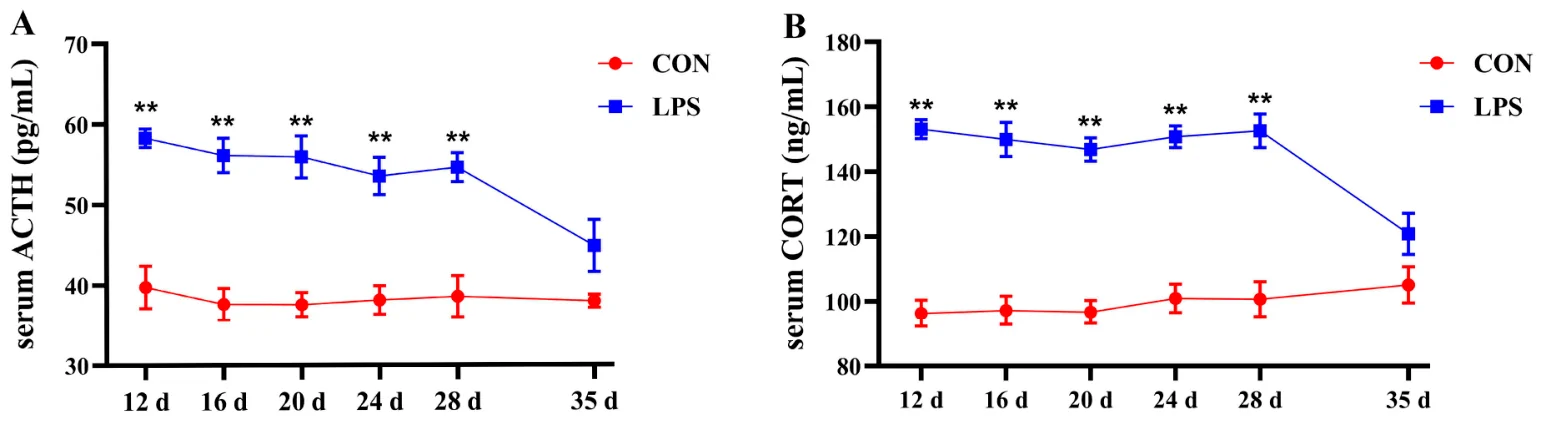
The effect of LPS injection on the levels of corticosterone and adrenocorticotropic hormone in the serum of broilers at different time stages. (**A**) Serum ACTH level. (**B**) Serum CORT level. The experimental animals were respectively treated with lipopolysaccharide (LPS) or normal saline (the control group, CON), and the serum hormone levels were measured at 12, 16, 20, 24, 28 and 35 days of age. ** *p* < 0.01.

The effects of repeated LPS challenge on serum ACTH and CORT concentrations are shown in Figure 3A,B. During the challenge period, serum ACTH and CORT concentrations were significantly higher in the LPS group than in the CON group on days 12, 16, 20, 24, and 28 (*p* < 0.01), indicating a marked neuroendocrine stress response following repeated LPS administration. On day 35, the differences in ACTH and CORT concentrations between the CON and LPS groups were no longer statistically significant, suggesting partial recovery after cessation of the LPS challenge.

In addition, as shown in Table 4, the incidences of diarrhea and mortality during 12 to 28 d were numerically higher in the LPS group than in the CON group (20.0% vs. 8.3% and 6.7% vs. 1.6%, respectively). These observations were consistent with the greater physiological burden associated with repeated LPS challenge. However, diarrhea incidence and mortality were considered supportive observations rather than primary criteria for model validation, which was mainly based on the sustained elevations in serum ACTH and CORT concentrations during the challenge period.

### 3.2. Effects of Glycyrrhiza Polysaccharides (GPs) on Growth Performance in Broilers

The effects of GP on broiler productive performance were presented in Table 5. GP supplementation significantly increased BW at 35 d (*p* = 0.010) and increased ADG during 1–35 d (*p* = 0.009). In contrast, LPS challenge significantly decreased BW at 35 d (*p* = 0.007) and reduced ADG during 1–35 d (*p* = 0.007).

### 3.3. Effects of Glycyrrhiza Polysaccharides (GPs) on Ileal Morphology and Goblet Cells

**Figure 4:**
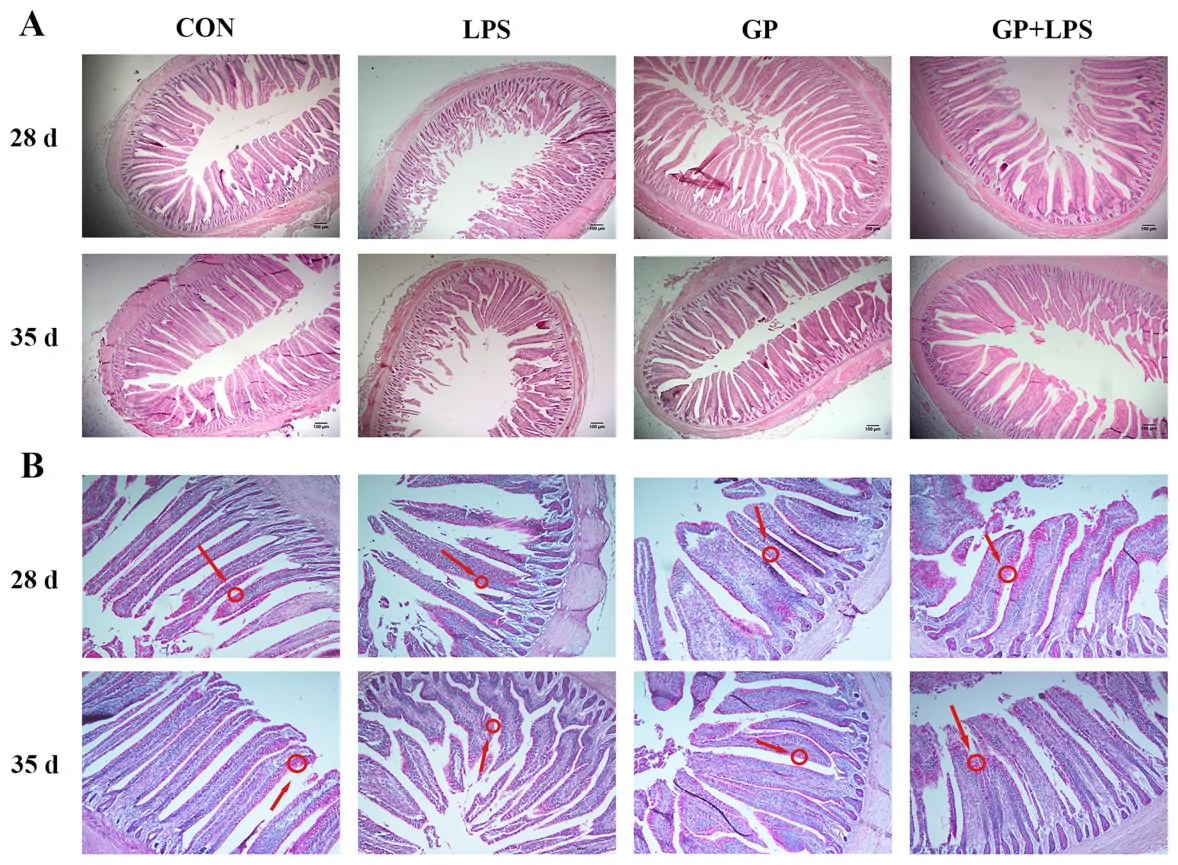
Effect of Glycyrrhiza polysaccharides (GPs) on morphological indices of the ileum in broilers. (**A**) H&E staining of the ileum at 28 and 35 days of age (HE staining, 40×). (**B**) PAS staining of goblet cells in the ileum at 28 and 35 days of age (PAS staining, 100×). Red arrowhead indicates goblet cells (red circle).

The effects of GP supplementation and LPS challenge on ileal morphology are presented in Table 6 and Figure 4. At 28 d, GP supplementation significantly increased VH (*p* = 0.004), VH/CD (*p* = 0.001), and GCs (*p* < 0.001); whereas, LPS challenge significantly decreased GCs (*p* < 0.001). At 35 d, GP supplementation significantly increased VH (*p* = 0.005), VH/CD (*p* = 0.001), and GCs (*p* = 0.017), while decreasing CD (*p* = 0.005). LPS challenge significantly decreased GCs at 35 d (*p* = 0.019).

### 3.4. Effects of Glycyrrhiza Polysaccharides (GPs) on Immune Organ Parameters in Broilers

The effects of GP supplementation and LPS challenge on relative immune organ weights are presented in Table 7. At 35 d, GP supplementation significantly increased the relative thymus weight (*p* = 0.005); whereas, no significant GP or LPS effects were detected for the relative weights of the spleen, bursa of Fabricius, or liver (*p* > 0.05).

### 3.5. Effects of Glycyrrhiza Polysaccharides (GPs) Supplementation and LPS Challenge on Serum Immune-Related Parameters in Broilers

The effects of GP supplementation and LPS challenge on serum immune-related parameters are shown in Table 8. At 28 d, significant GP × LPS interactions were observed for IL-4, IgA, IgG, and iNOS (*p* < 0.05). LPS challenge increased IL-4, IgA, IgG, and iNOS in birds fed the CON diet. In GP-supplemented birds, LPS decreased IL-4; whereas, no significant changes were observed in IgA, IgG, or iNOS. GP supplementation significantly decreased serum IL-1β, NF-κB, and TNF-α (*p* < 0.05). LPS challenge significantly increased IL-10, NF-κB, TNF-α, and IgM (*p* < 0.05).

At 35 d, significant GP × LPS interactions were observed for IL-1β, NF-κB, and sCD4 (*p* < 0.05). GP supplementation decreased IL-1β under non-LPS conditions, but no significant difference was observed between the LPS and GP + LPS groups. GP supplementation reduced NF-κB in LPS-challenged broilers. LPS challenge increased sCD4 under both dietary conditions. In addition, GP supplementation significantly increased IgA and decreased iNOS; whereas, LPS challenge significantly increased IL-6, IL-10, and TNF-α (*p* < 0.05).

### 3.6. Spleen Transcriptome

**Figure 5:**
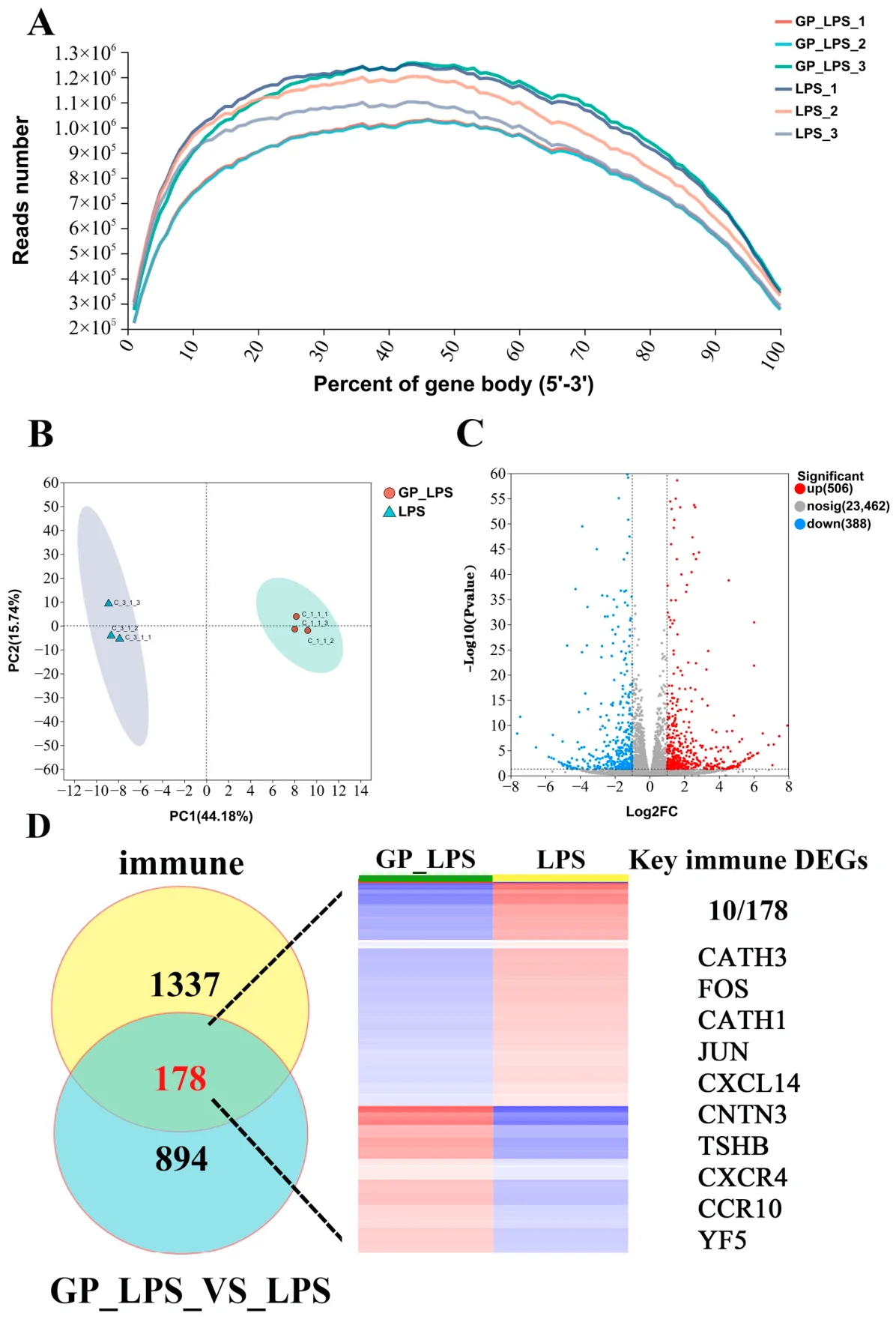
Transcriptome data analysis of broilers spleen. (**A**) Transcript coverage across the reference genome for each sample, indicating sequencing quality. (**B**) Principal component analysis (PCA) showing expression pattern differences between treatment groups. (**C**) Volcano plot of differentially expressed genes (DEGs) between the LPS and GP + LPS groups, with each point representing an individual gene. (**D**) Hierarchical clustering heatmap analysis of DEGs between the LPS and GP + LPS groups. Green and yellow represent the GP-LPS and LPS groups, respectively. Red color indicates that the gene is highly expressed in this sample, while blue color represents low expression.

Transcriptome sequencing was performed on the LPS stress model as a negative control and the GP + LPS group, as shown in Table 9. The original number of read segments for each sample ranged from 45.43 to 64.86 million. After quality control, the effective read segments were 44.98–64.16 million, and the proportion of valid data was all above 98.93%, indicating good data quality. The Q30 values of all samples ranged from 94.59 to 95.81%, and the GC content was 49.71–51.37%, It conforms to the typical characteristics of a genome and demonstrates the balance of the data. In order to further verify the reliability of the data, the sequencing coverage analysis graph is shown in Figure 5A, the curves in the graph do not show any obvious peaks, indicating that there is no bias in the sequencing process. Principal component analysis (PCA) (Figure 5B) indicates that clear separation among samples from different groups was observed, and the distribution within each group is extremely dense and compact, indicating distinct expression profiles between the LPS and GP + LPS groups. The volcano plot analysis further identified the DEGs, as shown in Figure 5C. Using a threshold of FDR < 0.05 and |log_2_FC| ≥ 1, altogether, 894 DEGs were identified after the GP intervention, among which 506 were upregulated and 388 were downregulated. A hierarchical clustering analysis of the immune-related DEGs between the LPS and GP + LPS groups is shown in Figure 5D. Genes sharing comparable expression profiles grouped into distinct clusters corresponding to each experimental group, demonstrating strong reproducibility across biological replicates. Key immune-related differential genes are displayed in the figure.

**Figure 6:**
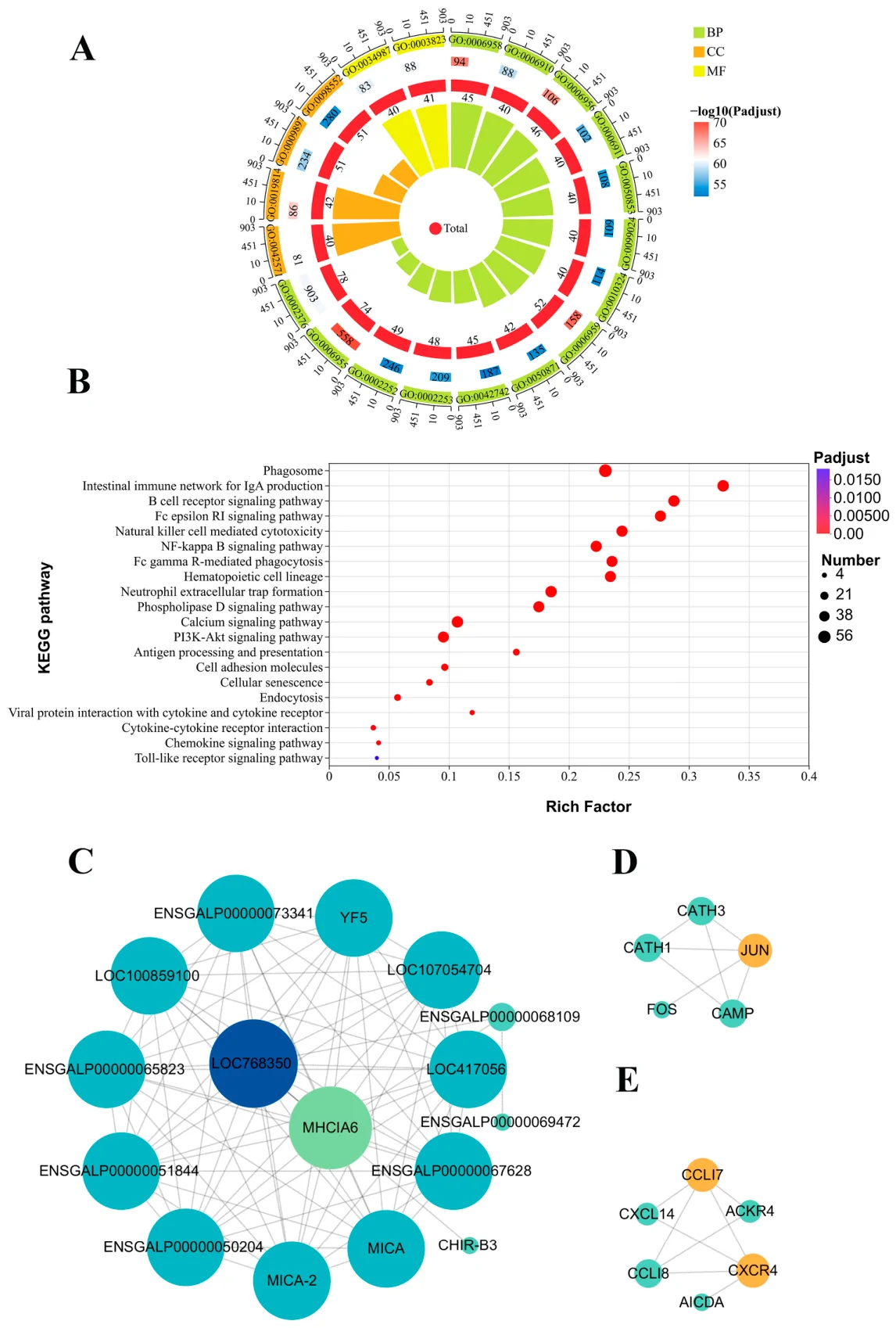
Differentially expressed genes (DEGs) in the spleens of broilers between the LPS and GP + LPS groups. (**A**) Multidimensional enrichment circle plot showing the enrichment of DEGs in Gene Ontology (GO) pathways. (**B**) Bubble plot showing the enrichment of DEGs in Kyoto Encyclopedia of Genes and Genomes (KEGG) pathways. (**C**–**E**) Protein–protein interaction (PPI) network diagram based on the coding of immune DEGs.

To investigate the functional roles of immunity-related DEGs, we carried out GO and KEGG enrichment analyses. The GO enrichment results are shown in Figure 6A, illustrating the enrichment of the three major functional categories. The twenty most significantly enriched GO terms are summarized as follows. In BP, the major enriched functions include immune response, humoral immune response, and complement activation. In CC, the enriched functions involve membrane side, immunoglobulin complexes, plasma membrane side, and circulating immunoglobulin complexes. In MF, the primary reinforcement functions are immunoglobulin receptor binding and antigen binding.

The results of KEGG pathway enrichment analysis are shown in Figure 6B. The top 20 significantly enriched pathways mainly focus on organism–environment interactions and immune response processes. Specifically, these pathways include phagosome, intestinal immune network for IgA production, B cell receptor signaling pathway, NF-κB signaling pathway, and phosphatidylinositol 3-kinase-Akt signaling pathway (PI3K/Akt), among others. Figure 6C–E. present the PPI network of immune-related DEGs, constructed using the STRING (v12.0) database, which provides an in-depth analysis of potential interaction patterns among these genes. Cytoscape (v3.10.3) software was used to visualize the results. The analysis revealed that 176 immune-related DEGs exhibited significant interactions, with major histocompatibility complex class I A6 (*MHCIA6*), Jun proto-oncogene (*JUN*), and C-X-C motif chemokine receptor 4 (*CXCR4*) identified as the core hub genes.

### 3.7. Validation of RNA-Seq Results Using qRT-PCR

**Figure 7:**
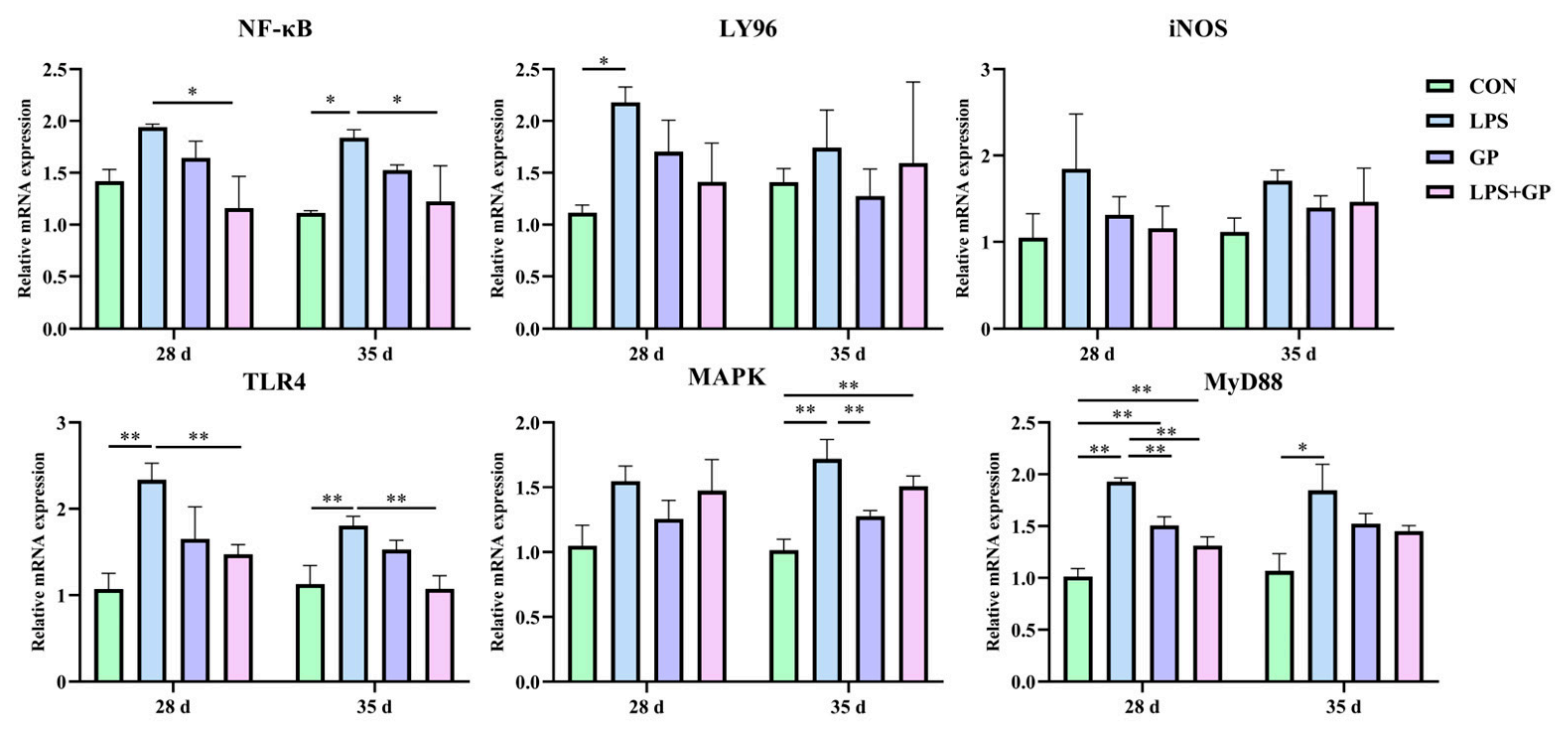
qRT-PCR gene expression data. The figure shows the relative mRNA expression levels of the MAPK inflammatory signaling pathway and related inflammatory factors in the spleen of broilers. Asterisks indicate statistical significance among treatments: * *p* < 0.05 and ** *p* < 0.01.

To assess the reliability of the RNA-Seq data, the expression levels of classic genes in the *NF-κB* signaling cascade were detected by qRT-PCR, as shown in Figure 7. The analysis revealed that under non-stress states over a period of 28 d, the addition of GP significantly elevated the gene expression level of *MyD88* (*p* < 0.05); under stress conditions, GP significantly alleviated the stress caused by LPS, namely the decrease in the expression levels of *NF-κB*, *TLR4*, and *MyD88* (*p* < 0.05). Under 35 days of stress conditions, GP significantly alleviates t LPS induced stress, that is, the expression levels of *NF-κB* and *TLR4* decrease (*p* < 0.05).

### 3.8. Spleen Proteome

**Figure 8:**
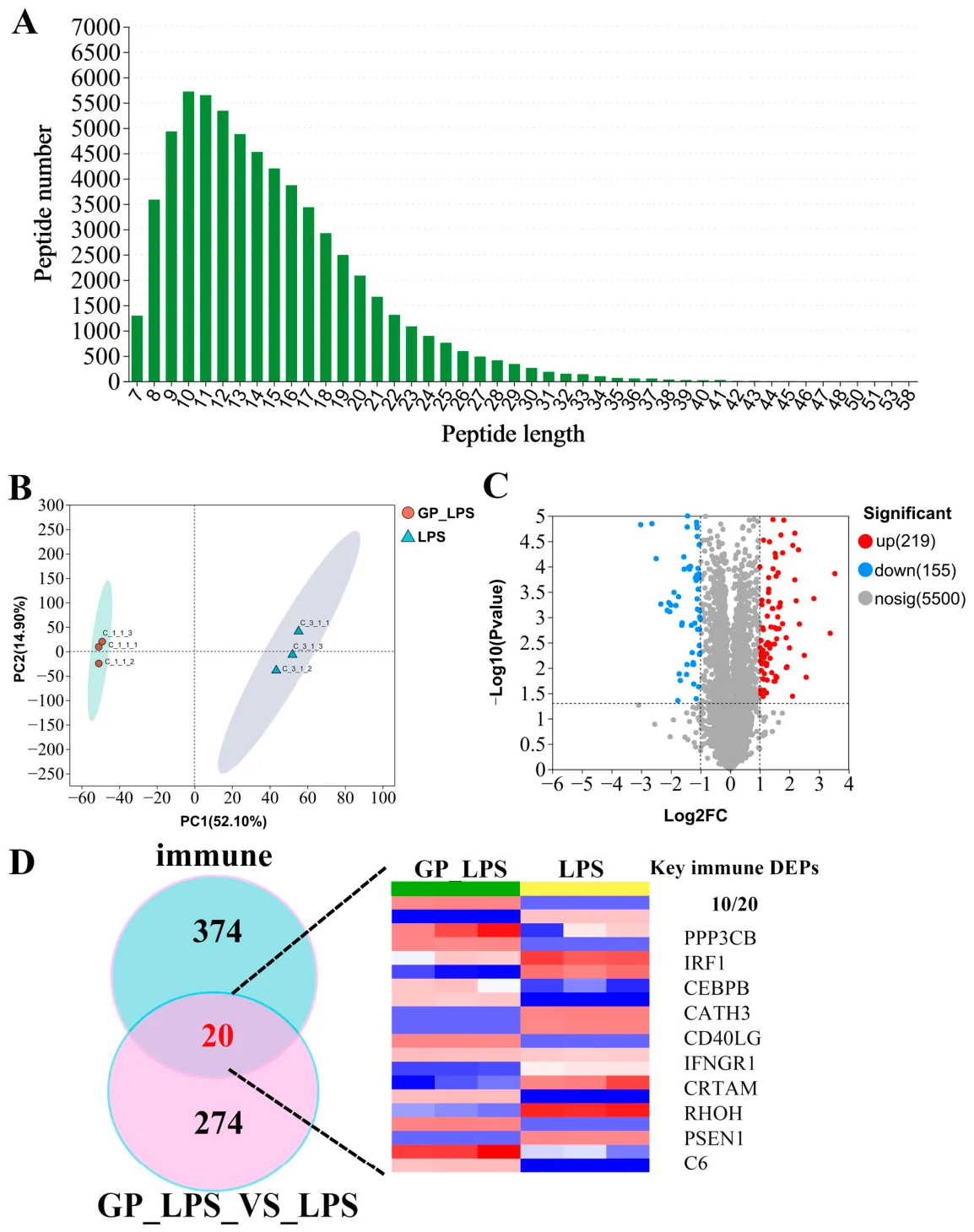
Proteomic analysis of broilers spleen. (**A**) Peptide length distribution plot of proteins. (**B**) Principal component analysis (PCA) showing expression pattern differences between treatment groups. (**C**) Volcano plot of differentially expressed proteins (DEPs) between the LPS and GP + LPS groups, with each point representing an individual protein. (**D**) Hierarchical clustering heatmap analysis of DEPs between the LPS and GP + LPS groups. Green and yellow represent the GP-LPS and LPS groups, respectively. Red color indicates that the gene is highly expressed in this sample, while blue color represents low expression.

This study further analyzed expression changes in immune-related proteins based on 4D label-free proteomics. The quality control results are shown in Figure 8A. The peptide lengths were predominantly distributed between 7 and 20 amino acids, consistent with standard proteomic analysis characteristics. These findings indicate thorough enzymatic digestion of samples and stable mass spectrometry detection, confirming satisfactory quality control results. PCA in Figure 8B demonstrates the homogeneity and heterogeneity between the two groups, revealing distinct protein expression profiles comparing the LPS and GP + LPS groups. These differences highlight the regulatory role of GP in the immune stress response. The volcano plot (Figure 8C) illustrates that, compared to the LPS group, GP intervention resulted in the identification of 374 DEPs. Among these, 219 proteins were significantly upregulated, while 155 were significantly downregulated, further supporting the intervention effect of GP. Figure 8D displays a clustering heatmap based on immune-related differential proteins. The heatmap results demonstrate significant differences in immune protein expression between the LPS group and the GP + LPS group, indicating that GP modulates immune protein expression and thereby mitigates the immune stress response induced by LPS.

**Figure 9:**
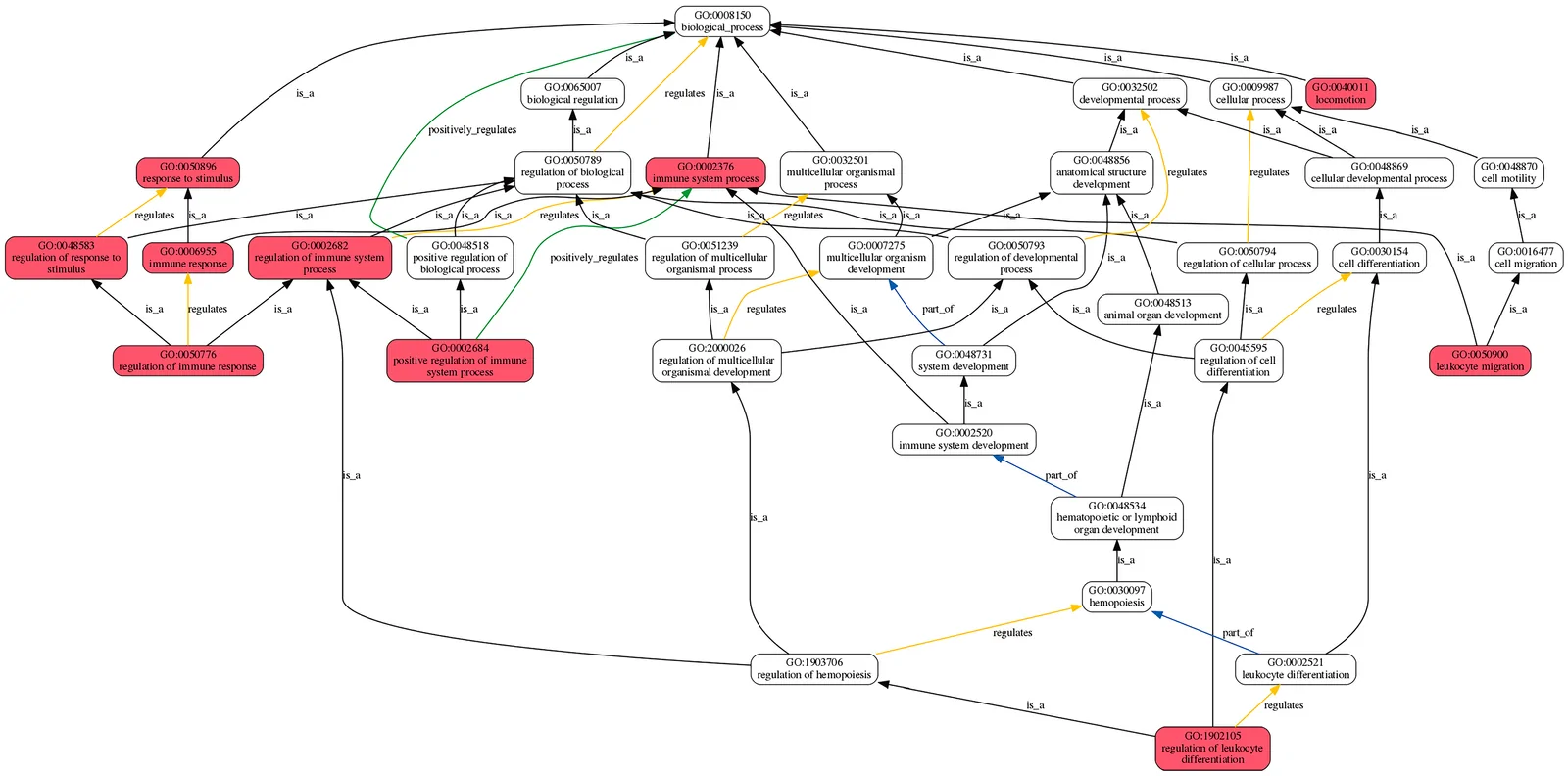
Directed acyclic graph (DAG) showing the enrichment of DEPs in GO pathways. Arrows indicate relationships between GO terms: black arrows represent is_a, blue arrows represent part_of, orange arrows represent regulates, and green arrows represent positively_regulates.

**Figure 10:**
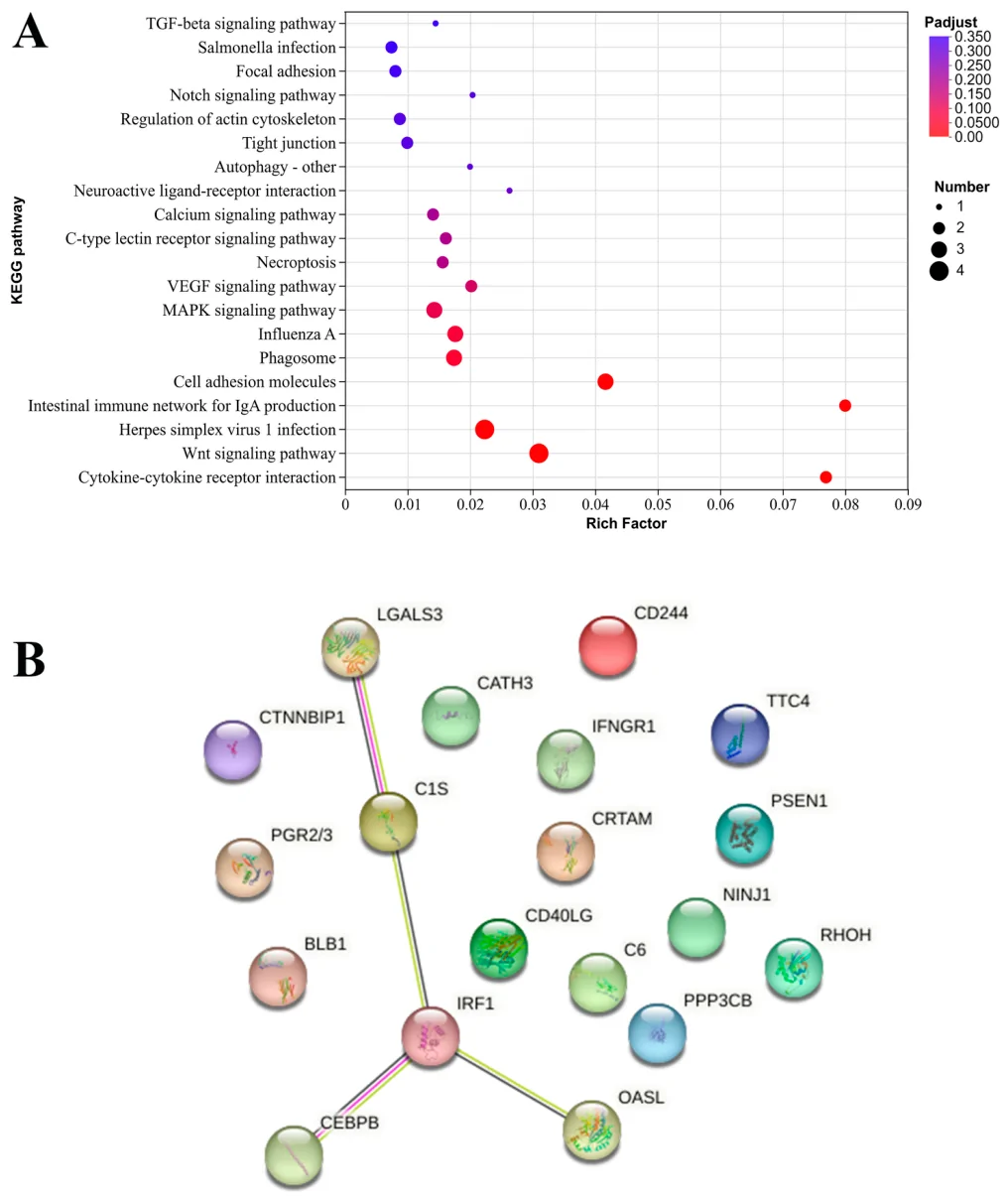
Kyoto Encyclopedia of Genes and Genomes (KEGG) pathway enrich-ment analysis of differentially expressed proteins (DEPs) in the spleens of broilers. (**A**) Bubble plot showing the enrichment of DEPs in KEGG pathways. (**B**) Protein–protein interaction (PPI) network diagram based on immune DEPs.

In this study, GO and KEGG enrichment analyses were conducted on the DEPs related to immunity comparing the LPS and GP + LPS groups. The relationships among various biological processes are systematically presented through a directed acyclic graph, as shown in Figure 9. The parts marked in red in the figure represent significantly enriched pathways. Among them, the immune system process (GO:0002376) serves as the core node and has the highest enrichment degree. This process is closely linked to the multicellular organism processes (GO:0032501), cellular processes (GO:0009987), and biological regulation (GO:0065007), and jointly participates in the regulation of biological processes (GO:0008150). Positive regulation of immune system process (GO:0002684) increases immune system activity by positively regulating the immune system process (GO:0002376). Meanwhile, the regulation of immune response (GO:0050776) acts as a regulatory mediator; by regulation of response to stimulus (GO:0048583), the regulation of immune system processes (GO:0002682), and the negative regulation of immune system (GO:0006955), it indirectly affects the response to stimulus (GO:0050896) and the regulation of biological processes (GO:0050789), demonstrating the multi-level regulatory role of the immune system in response to external signals. Furthermore, the GO entries related to the differentiation and migration processes of white blood cells were also significantly enriched. Among them, the regulation of leukocyte differentiation (GO:1902105) and leukocyte migration (GO:0050900) participated in the regulation of biological processes (GO:0008150) through the mediation of cellular processes (GO:0009987). KEGG revealed 15 highly enrichment-indicated routes (Figure 10A), primarily involving environmental responses and cellular signaling, including cytokine–cytokine receptor interactions, the Wingless (Wnt) signaling cascade, and the MAPK signaling pathway, all of which are considered to play pivotal roles in GP-mediated immune regulation. Finally, the interrelationship between immune-related differential proteins is shown through the PPI network diagram, as shown in Figure 10B.

### 3.9. Joint Analysis of Transcriptomic and Proteomic Data

**Figure 11:**
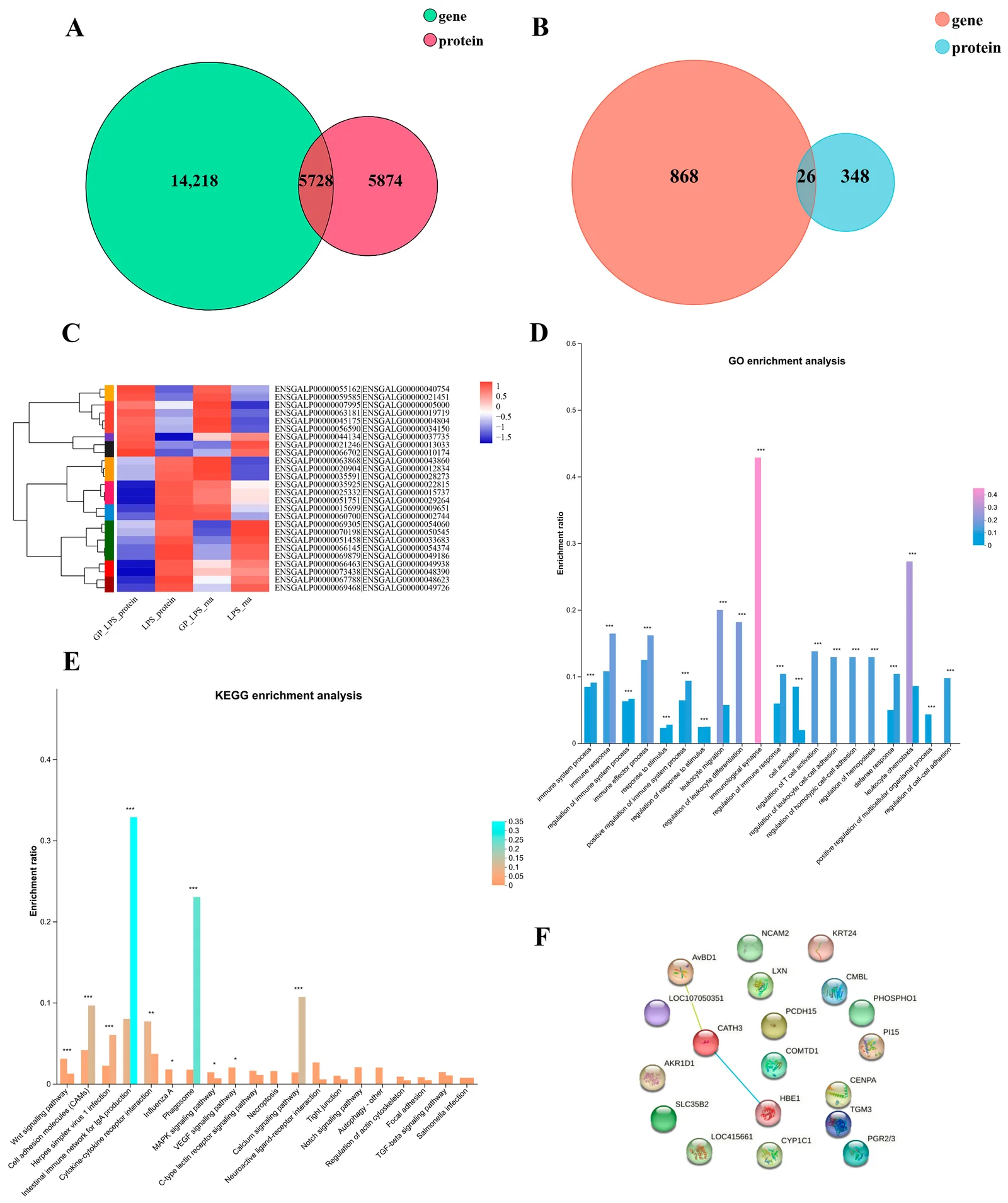
Integrated transcriptomic and proteomic analysis of LPS and GP + LPS groups in avian spleen. (**A**) The Venn diagram illustrates the total number of identified genes and proteins. The green circle represents genes, and the red circle represents proteins. The intersection indicates the overlap between genes and proteins. (**B**) The Venn diagram shows the differentially expressed genes and proteins. The blue circle represents differentially expressed genes, while the yellow circle represents differentially expressed proteins. (**C**) Hierarchical clustering heatmap analysis of differentially expressed gene–protein pairs. (**D**) Gene Ontology (GO) enrichment analysis presents the enriched functional categories based on differentially expressed genes and proteins. Column color gradients indicate enrichment significance. ***: *p* < 0.001; **: *p* < 0.01; *: *p* < 0.05. (**E**) Enrichment of differentially expressed genes and proteins in Kyoto Encyclopedia of Genes and Genomes (KEGG) pathways. Column color gradients indicate enrichment significance. ***: *p* < 0.001; **: *p* < 0.01; *: *p* < 0.05. (**F**) Protein–protein interaction (PPI) network analysis of differentially expressed gene-protein pairs.

Transcriptomic and proteomic data were integrated, utilizing the Ensemble database to standardize marker genes and protein identifiers, thereby minimizing potential biases in cross-omics integration. A total of 14,218 genes and 5874 proteins were included in the association analysis. Among these, 5728 gene–protein pairs exhibited significant correlations (Figure 11A). Figure 11B demonstrates that 868 DEGs and 348 DEPs were identified. Of these, 26 genes displayed differential expression at both transcriptional and translational levels. The cluster heatmap (Figure 11C) reveals that these 26 genes manifested consistent expression trends at the RNA and protein levels, with clear separation comparing the LPS and GP + LPS groups. Subsequently, GO and KEGG enrichment analyses were using the identified gene–protein pairs.

The GO enrichment results (Figure 11D) indicate that the primary enriched biological processes include immune system processes, immune responses, leukocyte migration, and cellular stimulation responses It indicates that the immune regulatory mechanism of GP involves multi-level regulation ranging from the molecular level to the systemic level. The KEGG enrichment analysis (Figure 11E) identified six significantly enriched pathways, including Wnt and MAPK signaling pathways, which provide insights into the functional roles of these genes and proteins in related biological pathways. A PPI network was constructed to further explore the interactions among the shared gene–protein pairs (Figure 11F). The analysis revealed that cathelicidin antimicrobial peptide 3 (CATH3) serves as a central node within the network, highlighting its potential pivotal role in specific biological processes.

## 4. Discussion

With the development of intensive broiler production systems, broilers are exposed to various immunogenic challenges during rearing, which may induce immune stress [26]. Previous work indicates that immune stress induces inflammatory responses that impair growth performance and compromise intestinal barrier integrity, thereby adversely affecting overall physiological development [27]. The results of the present study are consistent with previous findings, showing that LPS challenge significantly inhibited the growth performance of broilers. Specifically, reductions in ADG during days and 1–35, as well as in BW at 35 d, were observed, confirming the adverse effects of LPS-induced inflammation on broilers and supporting the successful establishment of the immune stress model. Previous studies have reported that plant-derived polysaccharides exert beneficial effects on growth performance and immune regulation in broilers [28]. In the present study, GP supplementation showed significant main effects on ADG and BW at 35 d, indicating a positive effect of GP on broiler growth performance, which is consistent with the findings of Zhao et al. [29]. However, no significant GP × LPS interaction was detected for growth performance under the conditions of this study. One possible explanation is that the growth-promoting effect of GP may occur primarily when immune homeostasis is maintained; whereas, under stress conditions, systemic inflammation induced by LPS challenge redirects nutrients toward maintenance of immune function rather than growth [30].

The intestine is the primary site for nutrient digestion and absorption and also serves as the body’s largest immune organ. Maintaining the completeness of the gut structure is crucial for effective immune defense [31]. The ileum is rich in Peyer’s plaques, which are components of intestinal-related lymphatic tissue and play an important role in mediating the intestinal immunoreaction [32]. Previous studies have reported that plant-derived polysaccharides can improve intestinal morphology [33,34]. In the present study, GP supplementation exerted significant main effects on ileal morphology, increasing VH, VH/CD, and GCs at both 28 and 35 d and decreasing CD at 35 d, consistent with previous reports. In addition, GP has been reported to promote goblet cell differentiation [35]. An increased number of goblet cells may enhance mucus synthesis and strengthen the mucosal barrier, thereby limiting pathogen adhesion and improving epithelial protection [36]. Overall, these findings indicate that GP contributes to the maintenance of ileal structural integrity and mucosal barrier function in broilers. In contrast, LPS challenge significantly reduced GCs at both sampling time points, indicating that LPS-induced inflammation impaired intestinal structure. However, no significant alleviating effect of GP on LPS-induced intestinal injury was detected. One possible explanation is that repeated LPS challenge induces systemic immune stress. Although GP can effectively regulate immune function, these immunomodulatory effects may not be accompanied by sufficient improvements in intestinal morphology to restore intestinal structure.

The relative weights of the liver, spleen, thymus, and bursa of Fabricius are widely used as morphological indicators of immune status [37]. In the present study, no significant effects of GP, LPS, or their interaction on immune organ parameters were observed at 28 d, possibly because the effects of GP or LPS on organ weights are time dependent. At 35 d, however, GP supplementation significantly increased the relative thymus weight. This effect may be related to the ability of GP to influence thymic development by promoting T-cell proliferation [38], thereby contributing to the alleviation of immune stress. This finding is consistent with the reported immunomodulatory properties of plant-derived polysaccharides [39].

Cytokines are the key indicators for measuring the immunity of broilers. Under inflammatory conditions, a range of cytokines encompassing proinflammatory mediators, such as IL-1β, IL-6, and TNF-α, and anti-inflammatory mediators, including IL-4 and IL-10, are released to maintain physiological homeostasis between inflammatory responses and immune regulation [40]. As a well-established ligand of TLR4, LPS activates NF-κB and other key transcription factors through MyD88- and TRIF-dependent signaling pathways, thereby inducing the production of multiple inflammatory cytokines [41]. In the present study, LPS increased serum NF-κB, TNF-α, IL-10, and IgM levels at 28 d, consistent with previous reports and indicating activation of NF-κB-related inflammatory signaling. GP supplementation significantly decreased the levels of the pro-inflammatory mediators IL-1β, NF-κB, and TNF-α, indicating an immunomodulatory effect of GP.

In addition, significant GP × LPS interactions were detected for IL-4, IgA, IgG, and iNOS at 28 d, indicating that GP participated in the regulation of responses to LPS-induced stress. IL-4 promotes Th2-cell differentiation and B-cell activation and proliferation, reflects humoral immune status, and is an important signaling molecule in adaptive immunity [42]. These findings suggest that GP may regulate the balance between inflammatory activation and immune homeostasis according to the physiological status of broilers. IgA and IgG are also important components of humoral immunity. IgA primarily strengthens the mucosal barrier and reduces the entry of pathogens through mucosal surfaces [43]; whereas, IgG mediates systemic humoral immune responses [44]. These results suggest that GP may alleviate LPS-induced inflammatory responses through modulation of adaptive immunity. iNOS is an important indicator of inflammatory status and reflects the physiological condition of the animal. GP supplementation significantly reduced iNOS levels, consistent with findings reported for other natural polysaccharides [45], further supporting the role of GP in alleviating LPS-induced inflammatory responses.

During the recovery phase, LPS challenge continued to exert significant main effects on several inflammatory mediators, including IL-6, IL-10, and TNF-α, indicating that the effects of LPS stimulation persisted after the challenge period. Meanwhile, GP significantly increased serum IgA and decreased iNOS levels, suggesting that GP continued to exert immunomodulatory effects during recovery by strengthening mucosal immune defense and reducing the inflammatory marker iNOS, thereby contributing to the maintenance of immune balance. Significant GP × LPS interactions were detected for IL-1β, NF-κB, and sCD4. IL-1β is a pleiotropic pro-inflammatory cytokine, and the observed interaction indicates that the regulatory effect of GP on IL-1β was dependent on the physiological condition of the birds. Under non-LPS conditions, GP significantly decreased IL-1β; whereas, no such reduction was observed in LPS-challenged broilers. This finding indicates that the suppressive effect of GP on IL-1β was not maintained during the recovery phase following repeated LPS challenge. This response may be related to differences in the temporal regulation of individual cytokines, although the underlying mechanism requires further investigation. NF-κB is a central regulator of immune and stress responses [46], and the present results indicate that GP significantly attenuated LPS-induced inflammatory activation during the recovery phase, thereby contributing to the maintenance of immune homeostasis. sCD4 is an immune marker associated with CD4+ T-cell activation, and elevated sCD4 levels reflect enhanced adaptive immune activation [47]. The reduction in sCD4 following GP supplementation suggests that GP may contribute to the restoration of immune balance during the recovery phase.

In cells, changes in mRNA levels provide the basis for protein expression, and proteins are the ultimate carriers of biological functions. Their expression and regulation often differ from those at the transcriptional level [48]. Although 26 genes were identified as differentially expressed in both transcriptomic and proteomic analyses, only 13 genes showed consistent changes in both aspects and were enriched in biological functions involved in immune responses and cell activation, suggesting that these immune-related processes play a crucial role in alleviating immune stress.

Under the immune stress state induced by LPS, excessive inflammatory response is one of the key reasons for the functional disorder of the body. Therefore, inhibiting inflammatory signals is regarded as an important prerequisite for alleviating immune stress [49]. Based on the significant changes in the NF-κB signaling cascade in the transcriptome data, this study selected the key genes in this pathway for qRT-PCR verification. Our results revealed that GP significantly alleviated the abnormal increase in the expression level of TLR4, NF-κB, and MyD88 induced by LPS, confirming that GP can mediate the inhibition of the excessive inflammatory response in the body and providing a basis for subsequent immune regulation and repair, thereby alleviating immune stress.

On the basis of controlling excessive inflammation, GP further affects the immune regulatory program. The transcriptome data show that in contrast to the LPS group, expression levels of JUN and Fos proto-oncogene (FOS) are upregulated and enriched in the MAPK pathway after GP supplementation. JUN and FOS interact to constitute the activator protein-1 (AP-1) transcription factor complex through heterodimerization, which is a key regulatory node in the body’s innate immune stage and participates in the precise modulation of immune-related gene transcription [50]. Meanwhile, the proteomic data show that interferon regulatory factor 1 (IRF1) is upregulated. It is known that IRF1 as an important transcriptional regulatory factor in the TRIF-dependent pathway, can participate in the regulation of MHC I genes and further regulate the antigen presentation process [51]. In line with this, the expression of MHCIA6 in the transcriptome was increased, suggesting that the antigen presentation function might have been enhanced [52]. Therefore, it can be inferred that GP does not merely suppress the inflammatory response; instead, it regulates the transition from the innate immune program to the adaptive immune response, thereby enhancing the overall immune level.

In addition to immune regulation, GP may also be involved in the recovery process after immune stress. Previous studies have shown that TLR4, in addition to activating downstream pathways through the MyD88 pathway, can also regulate the PI3K/AKT signaling pathway by recruiting PI3K [53]. In this transcriptome study, the expression of CXCR4 in the transcriptome was significantly upregulated after the addition of GP. Combined with previous studies that activated the CXCR4 receptor by adding Stichopus japonicus polysaccharides and initiated the PI3K/Akt signaling pathway, it can be seen that polysaccharides have the effect of inducing cell migration and alleviating stem cell damage [54]. Therefore, this evidence further supports that GP may be involved in the repair process that occurs after the body’s immune stress. Additionally, the PI3K/Akt signaling pathway can phosphorylate glycogen synthase kinase 3 beta (GSK-3β) via Akt, inhibit its activity, and enhance the stability of beta-catenin (β-catenin), thereby further regulating the Wnt signaling cascade [55]. In the proteome, catenin beta interacting protein 1 (CTNNBIP1) and presenilin 1 (PSEN1) were upregulated and enriched in the Wnt pathway. CTNNBIP1 acts as a binding protein for β-catenin and is a typical negative modulator of the Wnt pathway. It can prevent β-catenin from binding to the TCF/LEF complex, thereby inhibiting activation of Wnt signaling [56]. PSEN1 may further inhibit the activation of the Wnt pathway by enhancing the phosphorylation of β-catenin [57]. It has been reported that LPS excessively activates the Wnt signaling pathway, leading to abnormal cell proliferation. By adding shiitake mushroom polysaccharides, the stress induced by LPS can be alleviated [58]. GP can regulate the Wnt signaling pathway through negative feedback, preventing abnormal cell proliferation induced by LPS and maintaining immune homeostasis.

In the combined analysis, CATH3 was located at the core of the PPI network of differentially expressed proteins. It was significantly upregulated at both the transcriptome and proteome levels, indicating that CATH3 plays a central role in alleviating immune stress in GP. The cathelicidin antimicrobial peptide gene family plays a critical role in avian innate immunity, including CATH1, CAMP, and CATH3. Moreover, these three genes show upregulated expression in the transcriptome, suggesting that GP may regulate the overall expression level of the cathelicidin gene family [59]. Notably, the antibacterial effect of CATH3 is the most prominent among the members of this family. As a key target for GP to alleviate immune stress, it is speculated that GP regulates the activity of AP-1 by activating the MAPK signaling pathway, thereby influencing the expression of CATH3. This mechanism is consistent with previous studies [60].

## 5. Conclusions

**Figure 12:**
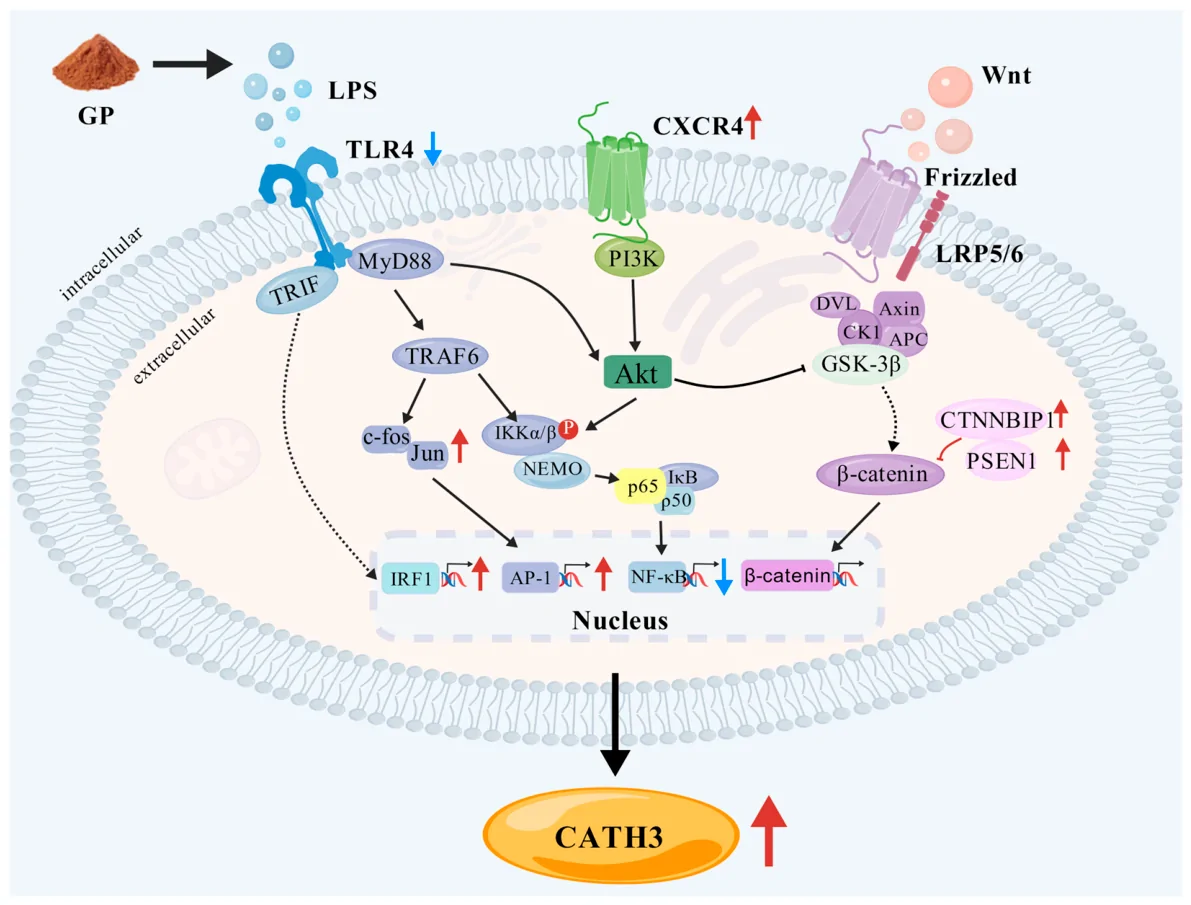
Regulatory effect of Glycyrrhiza polysaccharides (GPs) on LPS-induced immune stress in broilers. GP can inhibit the activation of TLR4 and its downstream MyD88 and TRIF-dependent signaling pathways, reducing the nuclear translocation of NF-κB. Additionally, GP activates the CXCR4-mediated PI3K/Akt signaling pathway and inhibits the activity of GSK-3β. Moreover, the upregulation of CTNNBIP1 and PSEN1 in the Wnt/β-catenin pathway will inhibit this pathway. The coordinated regulation of NF-κB, AP-1, IRF1 and β-catenin transcriptional activities will ultimately upregulate the expression of CATH3, thereby participating in immune regulation. In the figure, the red arrows: upregulation; the blue arrows: downregulation. The solid/ dashed black lines: direct/indirect regulation. Created with BioGDP.com [61].

In conclusion, GP can effectively alleviate the immune stress response in broilers induced by LPS. Specifically, it can inhibit the abnormal increase in inflammatory-related cytokine levels and optimize intestinal architecture. Furthermore, through the combined analysis of transcriptomics and proteomics, this study identified CATH3 as a key differentially expressed gene and protein, which may play a regulatory role in immune-related signaling cascades such as MAPK, PI3K/Akt, and Wnt by participating in the innate immune response and the regulation of cytokines (Figure 12). The above results support the use of GP as a new type of immunomodulatory feed additive for green poultry farming and offer a theoretical basis for its potential molecular basis in alleviating stress.

## Figures and Tables

**Table 1:** Vaccination protocol of AA broilers.

Item	7 d	14 d	20 d	24 d
Vaccine	Newcastle disease (ND) + Infectious bronchitis (IB) (LaSota + H120)	Infectious bursal disease (IBD) (B87 strain)	Infectious bursal disease (IBD) (B87 strain)	Newcastle disease (ND) + Infectious bronchitis (IB) (LaSota + H52)
Route	Ocular/Nasal instillation	Ocular/Nasal instillation	Ocular/Nasal instillation	Drinking water
Dosage	1 dose	1 dose	1 dose	1 dose

Note: One dose is defined as the standard amount of vaccine required for a single bird.

**Table 2:** Composition and nutrient levels of the basal diet (air-dry basis) unit: %.

Item	Feeding Phase	
	Day 1 to 28	Day 28 to 35
Ingredient (%)		
Corn	54.00	58.00
Peeled soybean meal	29.00	26.00
Flour	4.00	4.00
Corn protein powder	4.00	3.00
Soybean oil	2.25	2.80
Limestone	1.35	1.30
dicalcium phosphate	1.75	1.25
Hydrolyzed feather meal	1.00	1.00
Sodium chloride	0.25	0.30
Sodium humate	0.20	0.20
sodium bicarbonate	0.15	0.10
L-tyrosine	0.12	0.11
Choline chloride	0.10	0.10
DL-methionine	0.28	0.24
L-lysine	0.55	0.60
Premix ^1^	1.00	1.00
Total	100.00	100.00
Nutrient levels ^2^		
Metabolizable energy, (MJ/kg)	12.55	12.59
Available Phosphorus	0.43	0.40
Digestible Lysine	1.01	0.90
Digestible Methionine	0.31	0.29
Digestible Total Sulfur Amino Acids	0.61	0.57
Digestible Threonine	0.77	0.64
Digestible Tryptophan	0.21	0.19
Analyzed nutrient (%) ^3^		
Crude Protein	22.00	20.00
Calcium	1.00	1.00
Total Phosphorus	0.63	0.62

Note: ^1^ Provided per kg of diet: Vitamin A, 15,000 IU; Vitamin E, 47 IU; Vitamin K, 6 mg; Thiamine, 3 mg; Riboflavin, 9 mg; Pyridoxine, 6 mg; Cobalamin, 0.03 mg; Niacin, 60 mg; D-Pantothenic acid, 16 mg; Folic acid, 1.5 mg; Biotin, 0.06 mg; Choline, 900 mg; Zinc sulfate, 40 mg; Ferrous sulfate heptahydrate, 80 mg; Sodium selenite, 0.15 mg; Calcium iodate, 0.35 mg; Copper sulfate pentahydrate 8 mg, and manganese sulfate monohydrate 80 mg. ^2^ Calculated values. ^3^ Measured values.

**Table 3:** Primer sequence.

Gene ^1^	Gene NO.	Primer Sequence (5′−3′) ^2^	Fragment Size
β-actin	NM_205518.2	F: ATTGTCCACCGCAAATGCTTC	113 bp
		R: AAATAAAGCCATGCCAATCTCGTC	
iNOS	NM_204961.2	F: TCATCTGACCGTTGCAGTGGTA	117 bp
		R: AAGCACGGGACTGTTTCATTGAG	
NF-κB p65	D13721.1	F: CTATAAGTGTGAAGGGCGCTCAG	130 bp
		R: CGTCACCAAAGAGACCCGAAC	
TLR4	NM_001030693.2	F: CTGTACAGGGCTGGGCAAAG	148 bp
		R: GACAACCACAGAGCTCATGCATC	
MyD88	NM_001030962.5	F: ACTCACTCCTGGCTGGCTCA	129 bp
		R: CACGTGTCATGGCTGTTAATTCA	
LY96	XM_040696637.2	F: GGAGCTGGACATCCCAATGAA	89 bp
		R: TTTCCATAACTGAAGGCACAGCA	
p38-MAPK	NM_001043531.1	F: TGTGTTCACCCCTGCCAAGT	149 bp
		R: GCCCCCGAAGAATCTGGTAT	

Note: ^1^ β-actin = actin beta; iNOS = inducible nitric oxide synthase; NF-κB p65 = nuclear factor kappa B subunit p65; TLR4 = toll like receptor 4; MyD88 = myeloid differentiation primary response 88; LY96 = lymphocyte antigen 96; p38 MAPK = p38 mitogen-activated protein kinase. ^2^ F = forward primer; R = reverse primer.

**Table 4:** Diarrhea incidence and mortality rate of broilers subjected to immune stress induced by LPS.

Treatment	Age (d)	Broiler Count	Diarrhea Incidence (%)	Mortality Rate (%)
CON	12–28	60	8.3	1.6
LPS	12–28	60	20.0	6.7

Note: CON, control group. LPS, lipopolysaccharide challenged group. Diarrhea incidence and mortality rate were calculated based on the total number of broilers in each treatment during 12–28 d.

**Table 5:** Effects of Glycyrrhiza polysaccharides (GPs) on broiler growth performance.

Items	CON		GP		SEM	*p*-Value		
	−LPS	+LPS	−LPS	+LPS		GP	LPS	LPS × GP
BW (g)								
1 d	51.11	51.21	49.75	50.86	0.61	0.186	0.342	0.425
12 d	353.50	352.25	358.75	358.00	3.72	0.165	0.793	0.948
28 d	1063.45	1001.07	1080.58	1043.95	28.04	0.306	0.103	0.654
35 d	1502.03	1450.87	1602.98	1497.73	24.16	0.010	0.007	0.285
1–11 d								
ADG (g/d)	27.49	27.37	28.09	27.92	0.32	0.094	0.654	0.943
ADFI (g/d)	34.51	34.36	35.04	34.89	0.56	0.364	0.796	0.996
F/G	1.26	1.26	1.25	1.25	0.01	0.473	0.896	0.926
12–28 d								
ADG (g/d)	41.76	38.17	42.46	40.35	1.59	0.382	0.098	0.649
ADFI (g/d)	67.02	65.45	68.54	66.17	1.98	0.583	0.341	0.843
F/G	1.60	1.71	1.61	1.66	0.06	0.687	0.206	0.573
29–35 d								
ADG (g/d)	62.65	64.26	74.63	64.83	5.18	0.249	0.444	0.292
ADFI (g/d)	130.47	131.42	136.51	138.20	7.13	0.386	0.856	0.960
F/G	2.09	2.06	1.83	2.20	0.13	0.656	0.220	0.155
1–35 d								
ADG (g/d)	41.46	39.99	44.38	41.34	0.69	0.009	0.007	0.278
ADFI (g/d)	69.49	68.87	71.61	70.74	0.92	0.050	0.436	0.896
F/G	1.68	1.72	1.69	1.71	0.02	0.919	0.218	0.574

Note: BW, body weight; ADG, average daily gain; ADFI, average daily feed intake; F/G, feed-to-gain ratio; SEM, standard error of the mean. *p*-values for the main effects of GP and LPS and their interaction (GP × LPS) were obtained using two-way ANOVA.

**Table 6:** Effects of Glycyrrhiza polysaccharides (GPs) on the ileal morphology of broiler chickens.

Items	CON		GP		SEM	*p*-Value		
	−LPS	+LPS	−LPS	+LPS		GP	LPS	LPS × GP
28 d								
VH, (μm)	426.48	396.44	488.39	457.24	19.14	0.004	0.126	0.977
CD, (μm)	124.25	133.32	115.44	112.54	8.18	0.086	0.710	0.473
VH/CD	3.472	3.135	4.380	4.233	0.26	0.001	0.363	0.718
GCs, (n/r)	126.92	86.83	185.42	149.58	18.67	<0.001	<0.001	0.941
35 d								
VH, (μm)	435.09	408.10	497.49	467.49	16.55	0.005	0.081	0.932
CD, (μm)	165.49	188.69	110.26	129.46	15.73	0.005	0.151	0.905
VH/CD	2.788	2.232	4.717	3.792	0.40	0.001	0.055	0.663
GCs, (n/r)	180.00	157.63	229.75	183.63	12.99	0.017	0.019	0.399

Note: VH, villus height; CD, crypt depth; VH/CD, villus height to crypt depth ratio; GCs, goblet cell counts; SEM, standard error of the mean. *p*-values for the main effects of GP and LPS and their interaction (GP × LPS) were obtained using two-way ANOVA.

**Table 7:** Effects of Glycyrrhiza polysaccharides (GPs) on the immune organs of broiler chickens.

Items	CON		GP		SEM	*p*-Value		
	−LPS	+LPS	−LPS	+LPS		GP	LPS	LPS × GP
28 d								
RW of spleen	0.73	0.94	0.79	1.00	0.11	0.827	0.292	0.797
RW of Thymus	2.55	3.13	3.51	3.04	0.20	0.463	0.923	0.600
RW of Bursa	2.95	2.45	3.16	2.84	0.38	0.866	0.618	0.492
RW of Liver	27.80	31.05	28.55	27.18	1.73	0.789	0.894	0.931
35 d								
RW of spleen	0.78	0.78	0.91	1.01	0.07	0.100	0.200	0.351
RW of Thymus	3.07	2.97	4.00	3.99	0.28	0.005	0.982	0.876
RW of Bursa	3.06	3.88	3.42	3.30	0.24	0.575	0.155	0.087
RW of Liver	23.16	24.12	25.20	24.35	0.96	0.309	0.842	0.384

Note: RW, relative organ weight; relative organ weight = [organ weight (g)/body weight (g)] × 100; SEM, standard error of the mean. *p*-values for the main effects of GP and LPS and their interaction (GP × LPS) were obtained using two-way ANOVA.

**Table 8:** Effects of Glycyrrhiza polysaccharides (GPs) on the cytokines of broiler chickens.

Items	CON		GP		SEM	*p*-Value		
	−LPS	+LPS	−LPS	+LPS		GP	LPS	LPS × GP
28 d								
IL-1β (pg/mL)	178.66	185.87	115.66	107.32	18.84	0.005	0.977	0.691
IL-4 (pg/mL)	89.06 ^b^	143.34 ^a^	144.20 ^a^	102.58 ^b^	10.93	0.529	0.578	0.002
IL-6 (pg/mL)	197.93	280.02	162.53	256.98	27.82	0.586	0.050	0.643
IL-10 (pg/mL)	28.95	50.04	38.00	49.82	4.21	0.524	0.038	0.504
NF-κB (pg/mL)	941.63	1240.12	805.76	1014.43	38.22	0.001	<0.001	0.274
TNF-α (pg/mL)	39.60	53.55	24.70	35.91	3.57	0.007	0.009	0.712
IgA (μg/mL)	86.20 ^c^	231.21 ^a^	171.35 ^b^	198.53 ^ab^	18.16	0.186	0.001	0.012
IgG (μg/mL)	1108.36 ^b^	1737.83 ^a^	1154.48 ^b^	1304.04 ^b^	66.89	0.020	<0.001	0.007
IgM (μg/mL)	326.00	577.76	405.50	454.90	61.57	0.734	0.040	0.139
sCD4 (ng/mL)	4.96	8.32	9.21	8.63	0.53	0.149	0.360	0.206
sCD8 (ng/mL)	9.63	9.50	9.63	9.59	0.99	0.967	0.934	0.956
TLR4 (ng/mL)	14.86	17.22	14.02	14.88	1.81	0.408	0.401	0.691
iNOS (ng/mL)	14.82 ^b^	21.37 ^a^	17.44 ^ab^	15.78 ^ab^	1.29	0.308	0.109	0.016
35 d								
IL-1β (pg/mL)	165.84 ^b^	198.31 ^a^	116.14 ^c^	195.55 ^a^	8.66	0.016	<0.001	0.026
IL-4 (pg/mL)	118.60	125.47	113.18	84.45	10.48	0.057	0.327	0.127
IL-6 (pg/mL)	168.22	236.90	164.80	215.84	16.51	0.479	0.006	0.607
IL-10 (pg/mL)	42.70	55.57	39.46	49.94	3.43	0.231	0.009	0.783
NF-κB (pg/mL)	769.83 ^c^	1259.33 ^a^	714.58 ^c^	936.20 ^b^	45.16	0.003	<0.001	0.018
TNF-α (pg/mL)	36.58	54.40	35.31	45.81	4.35	0.290	0.011	0.425
IgA (μg/mL)	111.11	112.29	162.36	206.42	12.77	<0.001	0.114	0.132
IgG (μg/mL)	1412.9	1452.5	1351.0	1078.7	117.68	0.101	0.351	0.221
IgM (μg/mL)	461.45	466.00	600.12	465.98	44.03	0.154	0.179	0.154
sCD4 (ng/mL)	5.49 ^b^	9.51 ^a^	6.62 ^b^	8.45 ^a^	0.46	0.942	<0.001	0.047
sCD8 (ng/mL)	10.02	10.03	8.37	9.72	1.64	0.575	0.694	0.688
TLR4 (ng/mL)	15.93	15.31	14.98	14.64	1.46	0.598	0.752	0.928
iNOS (ng/mL)	15.56	15.55	13.16	11.61	1.13	0.023	0.510	0.512

Note: IL-1β, interleukin-1 beta; IL-4, interleukin-4; IL-6, interleukin-6; IL-10, interleukin-10; NF-κB, nuclear factor kappa B; TNF-α, tumor necrosis factor alpha; IgA, immunoglobulin A; IgG, immunoglobulin G; IgM, immunoglobulin M; sCD4, soluble cluster of differentiation 4; sCD8, soluble cluster of differentiation 8; TLR4, toll-like receptor 4; iNOS, inducible nitric oxide synthase; SEM, standard error of the mean. *p*-values for the main effects of GP and LPS and their interaction (GP × LPS) were obtained using two-way ANOVA. Different lowercase superscripts within the same row indicate significant differences among treatment combinations based on Tukey-adjusted multiple comparisons when a significant GP × LPS interaction was detected (*p* < 0.05).

**Table 9:** Transcriptome sequencing quantity statistics.

Sample	Raw Reads	Clean Reads	Valid Ratio (%)	Q30 (%)	GC Content (%)
GP + LPS	45,784,864	45,356,464	99.06	95.17	50.09
GP + LPS	45,431,210	44,989,040	99.03	94.59	50.25
GP + LPS	59,255,490	58,682,676	99.34	95.30	49.71
LPS	64,863,532	64,168,262	98.96	95.81	50.44
LPS	58,067,152	57,452,248	98.94	95.18	50.36
LPS	51,161,284	50,615,546	98.93	95.51	51.37

Note: GP + LPS, Glycyrrhiza polysaccharides (GP) supplementation combined with Lipopolysaccharide challenge. LPS, Lipopolysaccharide challenged group. Raw reads represent the total number of reads generated by sequencing. Clean reads represent reads obtained after quality filtering. Valid ratio represents the percentage of clean reads relative to raw reads. Q30 represents the percentage of bases with a Phred quality score ≥ 30. GC content represents the percentage of guanine and cytosine bases.

## Data Availability

Data generated in this study are publicly available. 1. The transcriptomic data have been deposited in the NCBI database under accession number PRJNA1303325 http://www.ncbi.nlm.nih.gov/bioproject/1303325 (accessed on 10 July 2026) 2. The proteome data were deposited in the iProX database under accession number PXD067169 https://proteomecentral.proteomexchange.org (accessed on 10 July 2026).

## Abbreviations

The following abbreviations are used in this manuscript:

GPs	Glycyrrhiza polysaccharides
LPS	Lipopolysaccharide
IL-1β	Interleukin-1 beta
IFN-γ	Interferon gamma
AA	Arbor Acres
H&E	Hematoxylin-eosin
PAS	Periodic Acid-Schiff
VH	Villus height
CD	Crypt depth
GCs	Goblet cell counts
IL-4	Interleukin-4
IL-6	Interleukin-6
IL-10	Interleukin-10
NF-κB	Nuclear factor kappa-B
TNF-α	Tumor necrosis factor-α
IgA	Immunoglobulin A
IgG	Immunoglobulin G
IgM	Immunoglobulin M
sCD4	Soluble cluster of differentiation 4
sCD8	Soluble cluster of differentiation 8
TLR4	Toll-like receptor 4
iNOS	Inducible nitric oxide synthase
CORT	Corticosterone
ACTH	Adrenocorticotropic hormone
DEGs	Differentially expressed genes
GO	Gene Ontology
KEGG	Kyoto Encyclopedia of Genes and Genomes
PPI	Protein–protein interaction
MyD88	Myeloid differentiation primary response 88
LY96	Lymphocyte antigen 96
p38 MAPK	P38 mitogen-activated protein kinase
SDS	Sodium dodecyl sulfate
TEAB	Triethylammonium bicarbonate
TCEP	Tris (2-carboxyethyl) phosphine
IAA	Iodoacetamide
TFA	Trifluoroacetic acid
DEPs	Differentially expressed proteins
PCA	Principal component analysis
PI3K/Akt	Phosphatidylinositol 3-kinase-Akt signaling pathway
MHCIA6	Major histocompatibility complex class I A6
JUN	Jun proto-oncogene
CXCR4	C-X-C motif chemokine receptor 4
Wnt	Wingless
CATH3	Cathelicidin antimicrobial peptide 3
FOS	Fos proto-oncogene
AP-1	Activator protein-1
IRF1	Interferon regulatory factor 1
GSK-3β	Glycogen synthase kinase 3 beta
β-catenin	Beta-catenin
CTNNBIP1	Catenin beta interacting protein 1
PSEN1	Presenilin 1
